# Digital Data Sources and Their Impact on People's Health: A Systematic Review of Systematic Reviews

**DOI:** 10.3389/fpubh.2021.645260

**Published:** 2021-05-05

**Authors:** Lan Li, David Novillo-Ortiz, Natasha Azzopardi-Muscat, Patty Kostkova

**Affiliations:** ^1^University College London (UCL) Center for Digital Public Health in Emergencies (dPHE), Institute for Risk and Disaster Reduction, University College London, London, United Kingdom; ^2^Division of Country Health Policies and Systems, World Health Organization, Regional Office for Europe, Copenhagen, Denmark

**Keywords:** digital data source, social media, healthier population, universal health coverage, health emergency, digital intervention

## Abstract

**Background:** Digital data sources have become ubiquitous in modern culture in the era of digital technology but often tend to be under-researched because of restricted access to data sources due to fragmentation, privacy issues, or industry ownership, and the methodological complexity of demonstrating their measurable impact on human health. Even though new big data sources have shown unprecedented potential for disease diagnosis and outbreak detection, we need to investigate results in the existing literature to gain a comprehensive understanding of their impact on and benefits to human health.

**Objective:** A systematic review of systematic reviews on identifying digital data sources and their impact area on people's health, including challenges, opportunities, and good practices.

**Methods:** A multidatabase search was performed. Peer-reviewed papers published between January 2010 and November 2020 relevant to digital data sources on health were extracted, assessed, and reviewed.

**Results:** The 64 reviews are covered by three domains, that is, universal health coverage (UHC), public health emergencies, and healthier populations, defined in WHO's General Programme of Work, 2019–2023, and the European Programme of Work, 2020–2025. In all three categories, social media platforms are the most popular digital data source, accounting for 47% (*N* = 8), 84% (*N* = 11), and 76% (*N* = 26) of studies, respectively. The second most utilized data source are electronic health records (EHRs) (*N* = 13), followed by websites (*N* = 7) and mass media (*N* = 5). In all three categories, the most studied impact of digital data sources is on prevention, management, and intervention of diseases (*N* = 40), and as a tool, there are also many studies (*N* = 10) on early warning systems for infectious diseases. However, they could also pose health hazards (*N* = 13), for instance, by exacerbating mental health issues and promoting smoking and drinking behavior among young people.

**Conclusions:** The digital data sources presented are essential for collecting and mining information about human health. The key impact of social media, electronic health records, and websites is in the area of infectious diseases and early warning systems, and in the area of personal health, that is, on mental health and smoking and drinking prevention. However, further research is required to address privacy, trust, transparency, and interoperability to leverage the potential of data held in multiple datastores and systems. This study also identified the apparent gap in systematic reviews investigating the novel big data streams, Internet of Things (IoT) data streams, and sensor, mobile, and GPS data researched using artificial intelligence, complex network, and other computer science methods, as in this domain systematic reviews are not common.

## Introduction

The proliferation of information technology has provided invaluable information and facilitated health communication among all users, generating staggering amounts of data (1). Digital data sources, such as social media and electronic health records (EHRs), offer a unique opportunity to study and understand health information, and impact population health. However, in addition to unquestionable opportunities, there are also access limitations and harmful impacts on health, which are often underestimated and under-researched.

Most of the previous systematic reviews have explored the effectiveness of specific sources on disease control or prevention, but there are few horizontal analyses and no systematic reviews of systematic reviews. To obtain a comprehensive understanding of digital data sources and their opportunities and impacts on public health, a systematic review of systematic reviews has been conducted through the lens of WHO's Thirteenth General Programme of Work (GPW13) (2) and its aligned, tailored workplan for the European Region, the European Programme of Work (EPW) 2020–2025 (3), highlighting three areas important for population health: universal health coverage (UHC), health emergencies, and healthier populations. According to WHO priorities, each of the three themes focuses on an essential aspect for global health populations, from primary health care to health promotion.

The objective of this systematic review is to understand the impact area of digital data sources on public health from published systematic reviews. More specifically, the aim is to obtain an overview of strength, good practices, and limitations of each digital source in each GPW13 and EPW core priority to explore their potential opportunities and offer appropriate recommendations to achieve its optimum utility.

The paper is structured as follows: in Section Data Sources and the GPW13 and EPW, we provide context by introducing the definition and classification of digital data sources on which this study focused and presenting the GPW13 and EPW defined by WHO and how we adapted them for this study; in Section Methodology, we outline the methodology for exploring and analyzing the existing reviews. Section Results describes the results by displaying the study characteristics and sorting out the key findings using data visualization tools. In Chapter 5, we discuss the challenges and provide suggestions on avenues for further research, and finally draw conclusions in Section Conclusion.

## Data Sources and the GPW13 and EPW

Data has always been instrumental for public health. For instance, upon collecting data on infections and deaths at a household level, John Snow helped control the spread of cholera in London's Soho region in 1854 (4) by pulling off the handle of the local water pump, proving that cholera is a waterborne disease rather than a “bad air” infection (5).

This section begins with traditional data sources created by medical professionals through health care IT patient management and reporting systems (electronic health/laboratory records) that have been traditionally embedded in public health surveillance, and then covers new data streams and big data generated by citizens, or user-generated data. Further, data sources also include mass media channels such as TV and radio. Data sources are not homogeneous (6). In this paper, we define the three groups of data providing the foundations for our systematic review of systematic reviews assessing their impact on citizens' health, in relation to the three priority areas defined by the GPW13 and EPW—UHC, health emergencies, and healthier populations.

### Data Sources

While traditional data sources collected by medical and health care professionals (such as EHRs) have been well-established since the boom of health informatics in the 1990s, in recent years, new data sources and solutions leveraged by artificial intelligence algorithms have dramatically enhanced health surveillance through data science advancements (7) and created a new, rapidly growing domain: digital epidemiology. This novel discipline utilizes traditional epidemiological data as well as novel digital data generated outside the public health system, that is, with data that were not generated with epidemiology as the primary purpose (8). Digital epidemiology is now an indispensable part of an established interdisciplinary domain of public health.

#### Electronic Health Records and Electronic Laboratory Reporting

**Electronic health records (EHRs)** are an invaluable source of clinical data capturing disease symptoms and medical treatments, and could be created in primary care settings (General Practitioner (GP) records), community health settings (health posts) and clinical settings (hospitals), or social and care facilities. They could be a helpful tool for public health surveillance and disease management [for e.g., the EHR-based surveillance system in New York City (9)], providing insights into previously unmonitored diseases. EHRs have the potential to add to the completeness of notifiable disease case reporting and enable longitudinal collection of cohort data related to specific diseases.

A wide variety of EHR data has been used in infectious disease surveillance, such as the incidence of Lyme disease and identifying newly diagnosed HIV infections (10), population health surveillance platforms (11), and outlining public benefits (accuracy, reliability, sensitivity, specificity, and timeliness) for communicable disease surveillance for de-identified personal EHR data (12). Klomps developed a platform called the Electronic Medical Record Support for Public Health (ESP), integrating EHR data for use in public health, while integrating clinical data into a repository for public health surveillance: the Public Health Community Platform (PHCP) (11).

**Electronic laboratory reporting (ELR)** has always been an essential part of patient diagnostic and specimen confirmation. It has also become critical to the disease surveillance process. In general, it refers to the automated transmission of reportable laboratory results from public health services, hospitals, and other laboratories to public health agencies. ELR also improves the effectiveness and efficiency of public health responses to outbreaks and cases of notifiable conditions. However, it often lacks the clinical information necessary to satisfy a case definition, such as symptoms, signs, and disease diagnoses (13). Hospital databases, including laboratory reports, are also an increasingly valuable source for surveillance. Surveillance systems provide real-time alerts to health care professionals (12).

To make the best use of EHRs/ELR for improving the health of citizens, we need better data accessing mechanisms and interoperability between systems to enable full access to health data by health authorities and free data from “health care information quarantine” to rapidly respond to emergencies like COVID-19 (14).

#### Mass Media

Traditional mass media include TV channels (broadcast or streamed online) and radio channels. Many public health campaigns before the era of the internet and social media (big data sources), or as a complement to big data sources, utilized mass media for health communication, health education, and raising awareness among populations and citizens.

#### Big Data Sources

Big data has become widely popular, although has many inconsistent definitions. For the purpose of this document, we define it by four dimensions (13, 14):

1. Volume—a large amount of information available.
2. Velocity of data acquisition, processing, and manipulation.
3. Variety of the data from different data sources and channels that can produce and release them.
4. Veracity or accuracy and reliability of the data collected.

Big data analytics refers to “the process of collecting, organizing, and analyzing large datasets, to discover patterns and generate useful, actionable information” (15), and require stream analysis to cope with the coming volume for real-time processing. The computer science discipline leveraged by big data is artificial intelligence (AI) spanning computer vision, machine learning, natural language processing (NLP), robotics, complex networks, and many more.

Observation of the spatio-temporal movements of millions of people during disease outbreaks (16), rapid detection of an unusual respiratory illness in a remote village anywhere on the globe (17), near real-time estimation of influenza activity levels (18, 19), using internet searches (18), and assessment of vaccination sentiments during pandemic preparedness efforts (20) are examples of opportunities for digital epidemiology (21–23) that exist because of the ability to reach out to and communicate with far more populations than ever before (24, 25).

##### Online News, Internet Media, and Websites

Online news and internet media resources are constantly covering public health events. Official media outlets, online newspapers, professional, and lay blogs, as well as personal home pages all became essential sources of early warning information for digital epidemiology. It was demonstrated that media coverage of a small outbreak appeared days before health authorities were informed about it through traditional surveillance processes (6).

While screening online news for specific diseases or conditions could help identify, for e.g., local food poisoning, robust epidemic intelligence (EI) systems screening all global media in multiple languages were developed to monitor epidemic events. These include a Health Canada-developed system called GPHIN (26)—a Joint Research Center (JRC)-funded monitoring system MediSys utilizing keyword extraction based on expert-defined weighted taxonomies, the comprehensive multiple data sources leveraging WHO Epidemic Intelligence from Open Sources (EOIS) system (27), and HealthMap (28), developed at Harvard Medical School.

##### Digital Traces, Online Searches, Sensors, and Internet of Things Devices

**Online searches** provide an invaluable, geolocation-enabled tool for monitoring public information needs that could reveal public sentiments, shopping panics, or disease outbreaks. This approach was first implemented by Google Flu Trends in 2008 and was followed by Google Dengue Trends (29). While there are enormous opportunities for mining search keywords by millions of users globally for public health use, the commercial ownership of the search logs by Google and other IT giants prevents researchers and public health experts from investigating this resource for public health purposes. In 2015, Google decommissioned the tools due to the lack of prediction accuracy (30).

Another opportunity for online search data was integrating EHRs and historical influenza-like illness data in models built on Google search terms, increasing accuracy of Google search trend data (31).

Online searches were also analyzed on public medical websites, such as the National electronic Library of Infection and National Resource for Infection Control (32–34) projects www.nric.org.uk, which is a unique example of the use of online searches from a non-commercial website to identify outbreaks. It should be noted, however, that information need spikes due to publication of major government guidelines (6, 35).

**Digital trace data** sources are generated by users searching the internet, using credit cards, travel cards, GPS-enabled phones, and any wearable and sensor devices, and collect information about our movements, physical locations, purchases, online preferences, and payments. They also include genomics data, imaging datasets, and data from sensors (36). For the sake of clarity, mobile phone-generated data and mobility (GPS) data are considered separately below. Location-aware applications leveraging personal data traces have revolutionized the way we travel, drive, navigate, and find local information in our everyday life, but also brought about a growing data privacy challenge.

Early warning of an upcoming outbreak of influenza could also be obtained by using mined payment card data to identify a spike in cough and flu medicine purchases. Payment and loyalty card data generated by consumer shopping behavior are more reliable than online search data in predicting a public health event. However, such datasets are typically owned by corporations (supermarkets, drugstores, etc.) and not available for research or public health purposes.

**Internet of Things (IoT)**-enabled devices and sensors allow streaming of real-time data readings and measurements. Attached to smart home devices, weather and pollution sensors, surveillance cameras, animal monitoring sensors, and human health monitoring sensors, IoT has revolutionized monitoring and enabled real-time analytics and a just-in-time response. However, IoT research still needs to leverage the opportunities created by IoT technologies for digital epidemiology, mapping the spread of infection (37).

##### Social Media Streams and Social Networks

Recent years have seen an unprecedented increase in user-generated content actively shared via social networking platforms such as Facebook and Twitter. Over the last decade, the popularity and proliferation of social networks have increased interaction among users, generating massive amounts of data on social media and offering a unique opportunity to study and understand social interactions as and when they happen (38).

While the privacy settings on Facebook, Instagram, and other social networks allow users to restrict access to their profile content and activity, the most important social media channel for research has undoubtedly become Twitter, due to its relatively open data policy allowing researchers and IT developers access to tweets through an open free application programming interface returning a 1% random sample of raw tweets free of charge (39). For this reason, Twitter has become a model organism for digital research data (40), providing an excellent way of sampling large populations and forecasting disease trends, monitoring emergencies, and gauging disease awareness and reactions to official health communications (41, 42).

Furthermore, Twitter is an efficient resource for tracking trends for the following reasons: (1) high frequency of posting allows real-time second-by-second analysis; (2) Twitter posts are more descriptive and publicly available; (3) Twitter datasets could be analyzed through either keyword search or natural-language processing techniques to identify relevance for researched topics, such as health conditions; (4) most tweets are geotagged, allowing development of spatio-temporal models of the spread of diseases or other dynamic phenomena based on human movement with great precision; (5) the Twitter user base is very broad, including populations from all generations; and (6) further insights could be revealed through analyzing demographic data and other details from user profiles and linking them to tweet content (43).

Twitter has been used to track (44, 45) and even predict (46) the spread of infectious diseases several weeks before they are identified or announced by public health authorities in the United Kingdom and the United States. Twitter data demonstrated the potential to extract spatio-temporal patterns to monitor outbreaks and their locations verified by correlations with the actual public health data. This research direction was pioneered for the swine flu pandemic in 2009 (44, 46–48) and for subsequent influenza outbreaks (49).

Twitter was demonstrated to be a powerful tool complementing traditional surveillance systems in terms of early warning and identification of virus subtypes in outbreaks (50). Twitter has been successfully used to predict influenza epidemics, with accuracy demonstrated by correlation with the ground truth, official surveillance data (51). In the 2011 *Escherichia coli* outbreak in Spain, tweets were analyzed to assess psychosocial factors in individuals and to implement food crisis communication strategies and monitor the population response (52). Twitter geolocation data have been successfully used alongside air traffic data to track the spread of Chikungunya virus (53) and have been incorporated into mechanistic models of influenza forecast intensity and outbreak peak time (54).

Twitter also plays a mixed role in terms of public information about vaccination for giving space to numerous anti-vaccination movements (55–57). Twitter has also been used in combination with other media to track scientific outreach at an international conference (58, 59).

Research on open profiles on other social media platforms, for e.g., Facebook investigating “like” features and Instagram timelines, has also demonstrated a correlation between health conditions and pandemic behaviors (40).

##### Mobile Data and GPS Mobility Data

Mobile phones and mobile apps have become indispensable in the twenty-first century. While the positive impact on mobile app data is demonstrated, the ownership and use of the data often restricted to the IT company that developed the app creates ethical challenges (60).

Mobile data also have the potential to improve digital surveillance. Effective mobile phone-based surveillance systems have been implemented in several countries with a high prevalence of a particular disease and a suboptimal surveillance system, producing moderate improvements in the completeness and timeliness of reporting [for e.g., eSurveillance implementation in the context of the Integrated Disease Surveillance and Response (IDSR) in the WHO African Region (61), influenza in Kenya (62), general syndromic surveillance projects in Papua New Guinea (63), malaria in Uganda (64), and Madagascar (65)].

A special role in the mobile datastreams is played by GPS location—mobility data—either collected and analyzed for locations and directions (navigation and mapping apps), or as a GPS-tag for other app-generated data (running apps). GPS location could also be extracted separately for contact tracing of infected cases during an epidemic. Several mobile location-aware technologies could be used for this purpose: Bluetooth, GPS, cellular location tracking, and QR codes.

For a contact tracing app to be effective, determining accurately the distance between two users (their mobile phones) requires precision of decimeters, even centimeters. For example, public health contact-tracing for COVID-19 defines a “contact” as a person who has been closer than 1.5 or 2 meters for at least 15 min. To improve accuracy, Bluetooth technology is used. The most important advantage of Bluetooth-based tracking solutions is that it could be completely user anonymous, thereby preserving privacy (66) and not collecting any personally identifiable user data or requiring data to be stored on a centralized server, which is not possible for GPS systems (67).

However, in terms of contact tracing effectiveness, even Bluetooth technology is not precise enough to avoid false positives, as the accuracy of determining the critical social distance of 1.5–2 m varies significantly depending on how people hold their phones, and whether they are indoors or outdoors (68–72).

These six data source categories will be explored in the systematic review:

1. Electronic health records (EHRs) and electronic laboratory reporting (ELR).
2. Mass media (MM).
3. Online news, internet media, websites (Web).
4. Digital traces, search engines (SE), sensors, and Internet of Things (IoT), including personal health devices.
5. Social media streams (SM) and social setworks (SNS).
6. Mobile data and GPS mobility data (Mobile/GPS).

In the next section, we cover the three priority areas of the GPW13 and EPW to set the scene for the systematic review.

### The GPW13 and EPW

**Figure 1:**
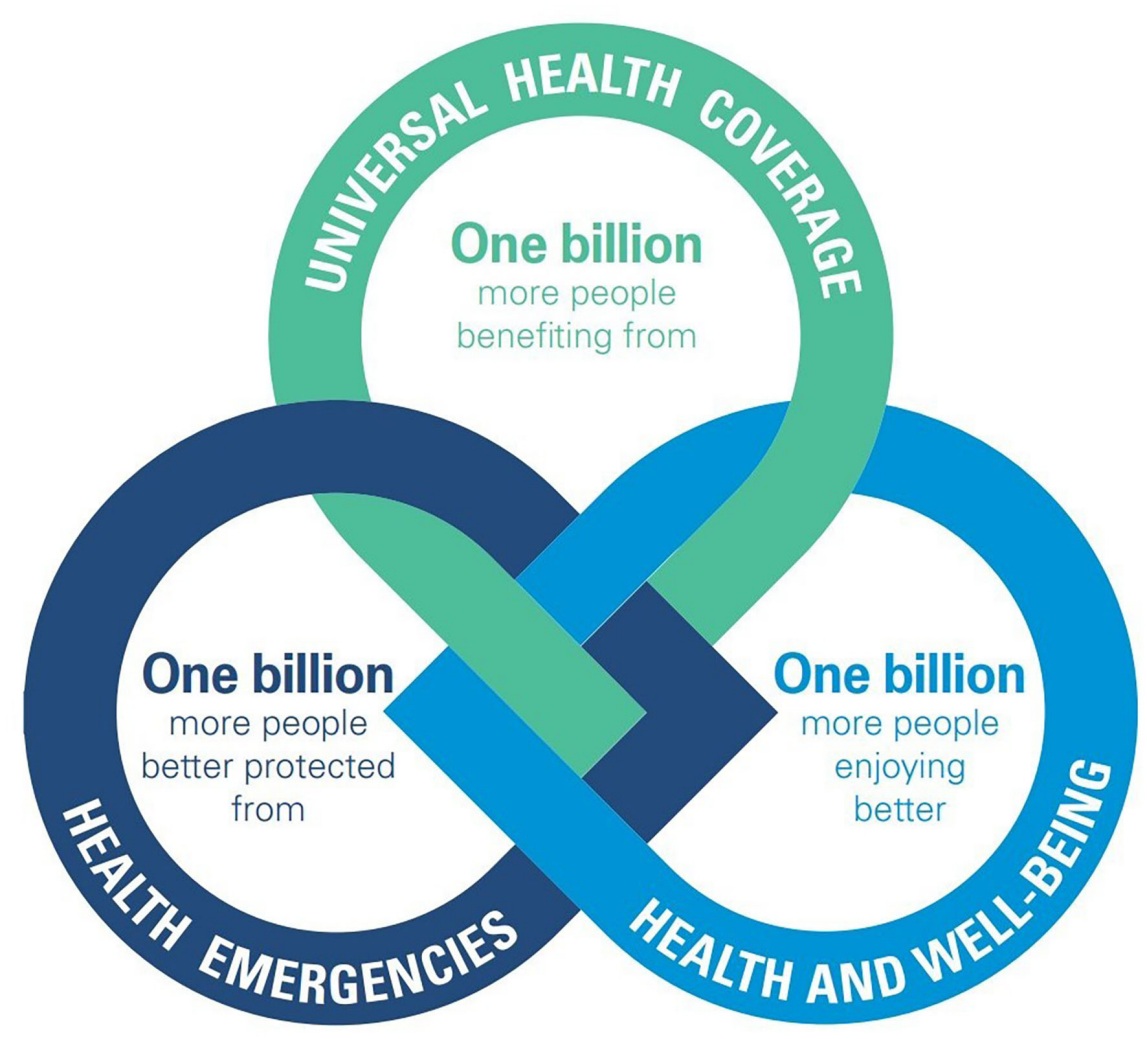
The GPW13: a set of interconnected strategic priorities and goals to ensure healthy lives and promote well-being for all people at all ages. Source: GPW13 (2).

WHO's Thirteenth General Programme of Work (GPW13) (2) focuses on measurable impacts on people's health at the country level, while its aligned and tailored workplan for the WHO European Region, the European Programme of Work (EPW) (3), sets out a vision of how the WHO Regional Office for Europe can better support countries in the region in meeting their citizens' expectations about health. As shown in Figure 1, we define the three areas as outlined in the GPW13 and EPW. We are investigating how the data sources defined above can improve and have measurable impacts on people's health.

#### Universal Health Coverage

In line with the definition set out in the GPW13, universal health coverage (UHC) asserts that all individuals and communities receive the health services they need without suffering financial hardship. The essence of UHC is universal access to a strong and resilient people-centered health system with primary care as its foundation (73). It includes the full spectrum of essential, quality health services, from health promotion to prevention, treatment, rehabilitation, and palliative care.

More specifically, as shown in Table 1, WHO uses 16 essential health services in four categories as indicators of the level and equity of coverage in countries.

**Table 1:** UHC indicators (73).

Reproductive, maternal, newborn, and child health (RMNC)	• Family planning • Antenatal and delivery care • Full child immunization • Health-seeking behavior for pneumonia
Infectious diseases (IF)	• Tuberculosis treatment • HIV antiretroviral treatment • Hepatitis treatment • Use of insecticide-treated bed nets for malaria prevention • Adequate sanitation
Non-communicable disease (NCD)	• Prevention and treatment of raised blood pressure • Prevention and treatment of raised blood glucose • Cervical cancer screening • Tobacco (non-)smoking
Service capacity and access	• Basic hospital access • Health worker density • Access to essential medicines • Health security: compliance with the International Health Regulations

Based on these definitions, we extracted a set of keywords to be included in the search (see Appendix 1) and classified the studies reviewed according to this category (Supplementary Table 1).

#### Health Emergency

It was demonstrated in the GPW13 (2) and EPW (3) that the priority of the health emergency strategy is to build and sustain resilient national, regional, and global capacities required to keep the world safe from epidemics and other health emergencies, and ensure that populations affected by acute and protracted emergencies have rapid access to essential life-saving health services, including health promotion and disease prevention. Therefore, in this group, our primary concerns are infectious diseases and outbreaks, as well as vaccines, which are critical for reducing potential risk. Consequently, emergency, pandemic/epidemic, and vaccine-related phrases are our keywords to detect the relevant systematic reviews in our search strategy, as shown in Appendix 1.

#### Healthier Populations

Based on the GPW13 (2), the planning of a healthier population is mainly based on the following criteria: the challenges they address erode the prospect of healthy lives, require a multisectoral approach addressing health determinants, represent existential threats to human flourishing, have associated opportunity costs amounting to trillions of dollars, and are areas where WHO has a comparative advantage. Its purpose is to contributte to people enjoying better health and well-being. Although that is admittedly a broad definition, it will be pursued through five platforms: (1) improving human capital across the life course, (2) accelerating action on preventing non-communicable diseases and promoting mental health, (3) accelerating elimination and eradication of high-impact communicable diseases, (4) tackling antimicrobial resistance, and (5) addressing health effects of climate change in small island developing states and other vulnerable states (2).

**Figure 2:**
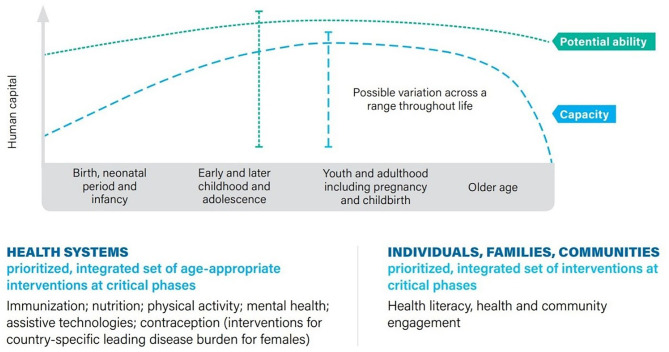
Increasing human capital throughout life through an integrated set of evidence-based interventions. Source: GPW13 (2).

For the purposes of this systematic review, we have extracted a set of keywords best representing the healthier population platforms in terms of access to or impact of data sources. Since these interconnected platforms also overlap with the other two priorities of UHC and health emergencies, to reduce the risk of bias, we simplify it into several age-appropriate items and evidence-based interventions, as shown in Figure 2. Our keywords in the healthier populations' category (see Appendix 1) are adapted from this figure, which is also used to classify the reviews collected (Supplementary Table 3).

## Methodology

**Figure 3:**
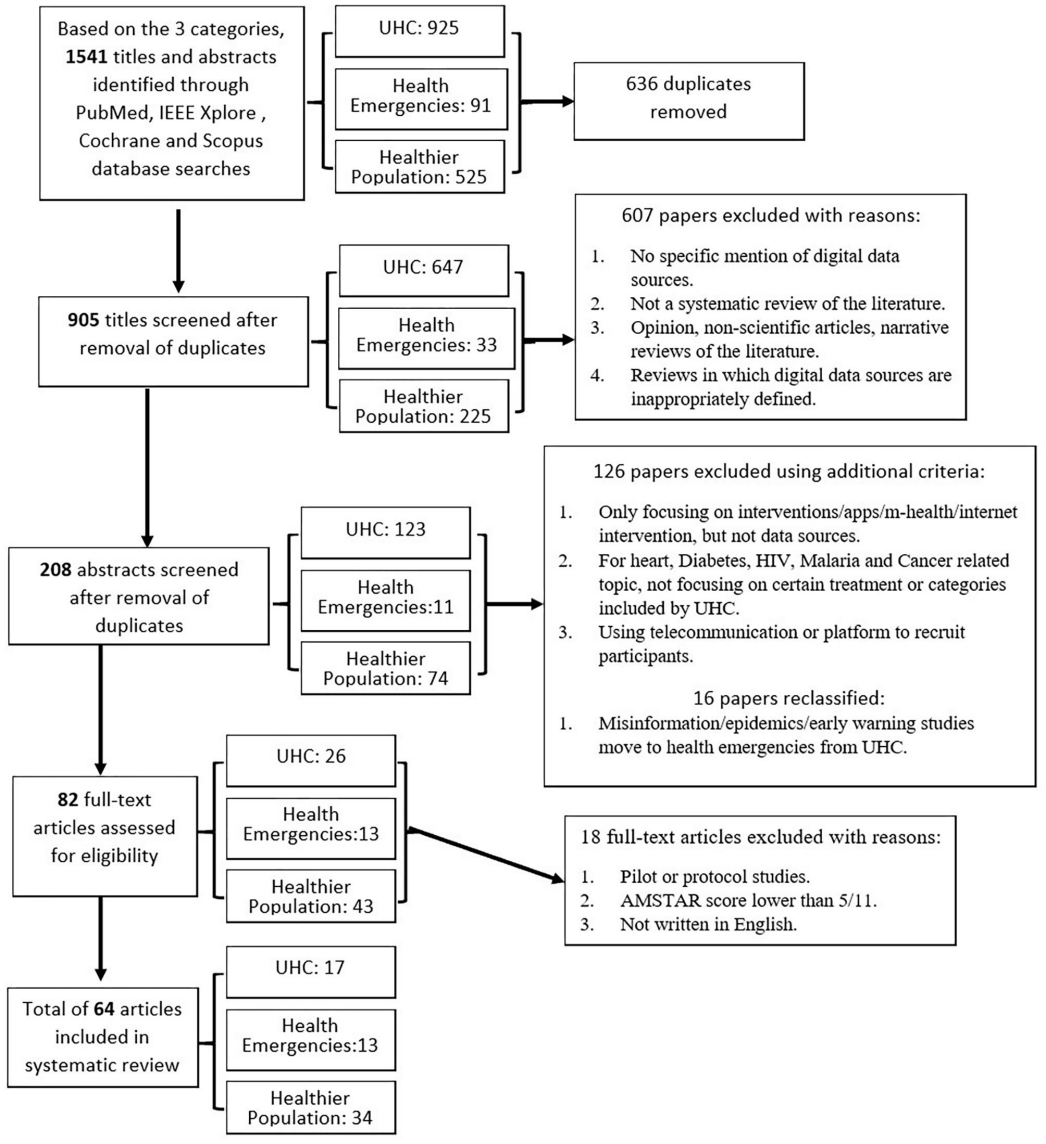
PRISMA diagram detailing the study identification and selection process.

A systematic review approach was adopted to search for systematic reviews from the four major research library databases: Scopus, IEEE Xplore, Cochrane, and PubMed. PRISMA (Preferred Reporting Items for Systematic Reviews and Meta-Analyses) selection (74) of reviewed systematic reviews was performed using exclusion and inclusion criteria and a process flow chart, seen in Figure 3. Based on the three categories, articles were selected using the predefined keywords and selection criteria. A total of 1,514 citations were identified, of which 64 unique studies met the criteria for inclusion; 17 topics were classified under universal health coverage (UHC), 13 studies under health emergencies, and 34 under healthier populations.

### Search Strategy

Study procedures followed PRISMA guidelines (74). We conducted a comprehensive literature search in online databases, including Scopus, IEEE Xplore, Cochrane, and PubMed. Advanced title, keyword, and abstract searches were performed for the publication date of 2010 and onwards to identify the systematic reviews in which digital data sources combined with each category (see Appendix 1).

### Inclusion and Exclusion Criteria

To narrow down the papers selected for the review, the following inclusion and exclusion criteria were used:

Inclusion criteria:

1. Studies provide an explicit link between digital data sources and their impact on people's health; the measurement of the impact could be qualitative (identifying the impact area) or quantitative (assessing the degree of influence).
2. Studies should be systematic reviews rather than opinions, non-scientific articles, or narrative reviews.
3. Literature published in English or Spanish.
4. Research subjects should fall under the three categories specified.
5. Studies show the challenges, opportunities, or good practices concerning digital data sources.

Exclusion criteria:

1. No specific mention of digital data sources.
2. Not a systematic review of the literature.
3. Studies contain inadequate information on the research methodology.
4. Unpublished studies.
5. The digital data sources are inadequately defined.

We have also assessed the reviews based on the additional exclusion criteria below:

1. Excluding studies that focus only on interventions/apps/mHealth/internet intervention, but do not refer to any data sources.
2. For heart-, diabetes-, HIV-, malaria-, and cancer-related topics, not focusing on specific treatment or categories included by UHC, then excluded.
3. Excluding studies using telecommunications or platforms to recruit participants for health studies.
4. Misinformation/epidemics/early warning studies should move to the health emergencies section unless they focus on compliance with the International Health Regulations.

### Data Extraction

The relevant articles were studied and the data were extracted at different stages. One researcher completed the data extraction and another researcher evaluated the studies for consistency with the aim and inclusion criteria, and disagreements were resolved by consensus.

### Quality Assessment

To obtain a valid estimate and reduce the risk of bias in the meta-analysis, we used the Assessment of Multiple Systematic Reviews checklist (AMSTAR) (75) to assess the methodological quality of the studies included. The AMSTAR criteria checklist (see Appendix 2) is composed of 11 reporting domains of published reviews. Each item is allocated 1 point; therefore, the highest score is 11 if all criteria are met, and scores of 5 and above are regarded as satisfactory. In this process, full-text articles were read and screened to assess the quality comprehensively.

### Study Synthesis

As defined in Section Data Sources and the GPW13 and EPW, the categories of digital data sources and the abbreviations used in this review are as follows:

**EHRs**: electronic health records.
**MM**: mass media.
**Web**: online news, internet media, websites.
**SE**: search engines.
**SM/SNS**: social media streams and social networks.
**Mobile/GPS**: mobile data and GPS mobility data.

In addition to categorizing the data sources, a comprehensive matrix of criteria was developed to indicate their impact on specific aspects of personal or population health and how the data sources were used to achieve a health outcome. The categories used and their abbreviation to assess and label the systematic reviews are defined below:

1. Aspects of personal or population health
  **PRM:** Using a digital data source to prevent or treat disease, including controlling the symptom or managing the records.
  **CH:** Digital data source is the cause of a disease or has a harmful impact on people's health.
  **AKB:** Having a positive impact on changing people's attitude, knowledge, and behavior to better know and control the disease, including vaccine uptake.
  **EW**: Using a digital data source as a tool to provide early warning for outbreaks.
  **PC**: Enhancing patient–clinician communication, increasing satisfaction with health care.
2. How the data sources were used:
  **Access**: The data sources were accessed by researchers (for e.g., EHRs via the web, phone).
  **Mining**: Using data mining to collect data from crowdsourced data sources.
  **Intervention**: Providing a platform for a digital intervention and the sampling data from participants were collected by researchers.

## Results

### Study Characteristics

Supplementary Tables 1–3 provides an overview of the characteristics of the studies meeting the criteria of the review (*N* = 64). There were 1 836 studies in total across all reviewed articles. The research was divided into three categories based on the GPW13 priorities (2) defined by WHO, that is, 17 in universal health coverage, 13 in health emergencies, and 34 in healthier populations, reflecting the overall impact of digital data sources on public health. Although a few reviews included studies in low-middle income countries like China, India, and Thailand, most of the studies are in developed countries such as the United States (*N* = 30), Australia (*N* = 18), the United Kingdom (*N* = 10), and Canada (*N* = 6). The studies included were heterogeneous in their design and outcome reporting, preventing the pooling of findings. As a result, outcomes and study details have been collated using tables and described narratively according to category, outcomes and limitation assessed, and data source components, including targeted group, AMSTAR score (75), and the number of studies assessed.

### Key Findings

In this section, we analyze the results of the systematic review according to the three GPW13 priority areas (2) defined by WHO. In each area, digital data sources have had various impacts on different targeted groups. By quantifying the number of reviews and their proportion, we can obtain a big picture of the influence of digital sources on human health in society as a whole. We also summarize and expound the strengths and limitations of digital sources in all three categories.

#### Data Sources and Universal Health Coverage

As summarized in Supplementary Table 1, in the UHC field, the reviews on the effects of digital data sources mainly focused on non-communicable diseases. Although several reviews explore its function in the HIV antiretroviral treatment (76) and delivery care (77–80), which belong to infectious disease and reproductive health groups, respectively (73), 14/17 studies relate to promotion of non-smoking and debate treatment. However, in the service and access group, no systematic reviews were found.

**Figure 4:**
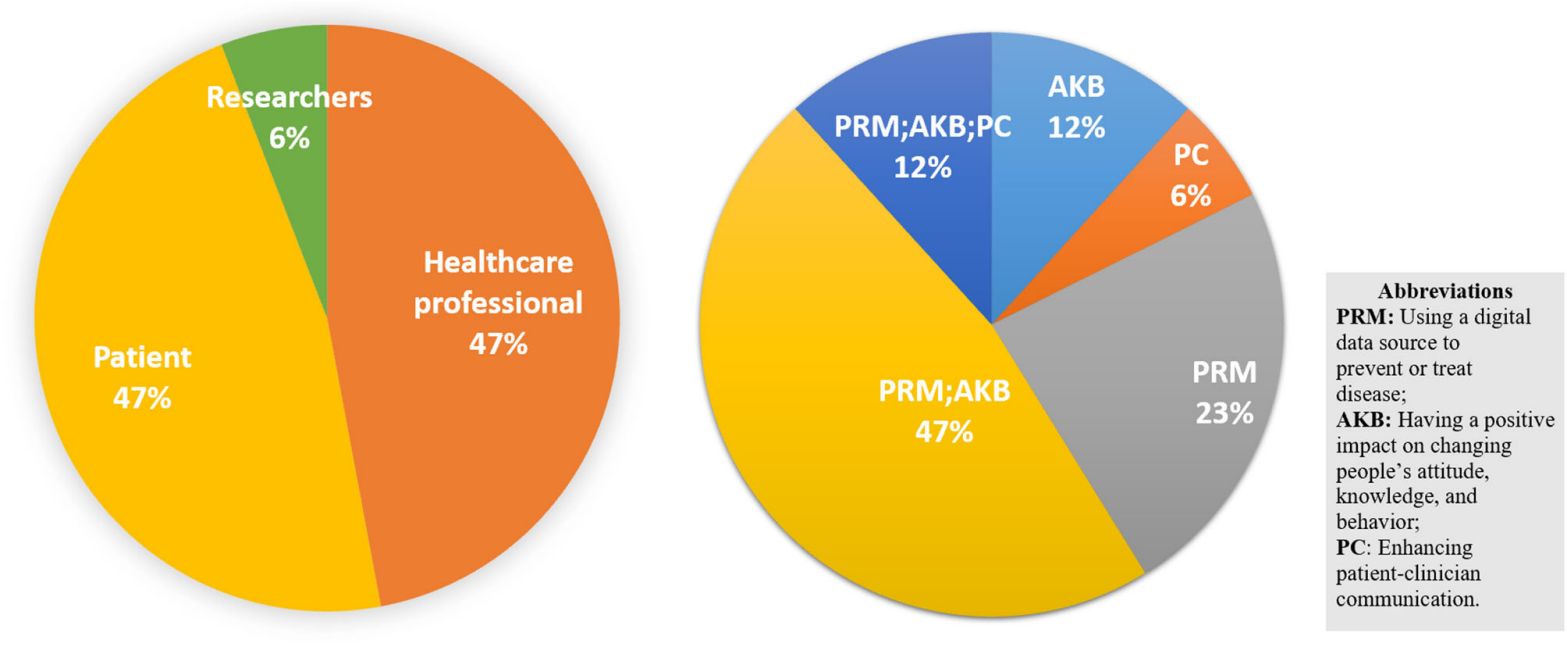
Pie chart of the targeted group (left) and impact (right) of digital data sources on UHC.

Most of the studies we found are provided for health care professionals (81–85) and patients (77, 79, 86–90) to manage disease or develop interventions through changing attitudes, behaviors, and knowledge to prevent potential health hazards. As shown in Figure 4, only 6% of the studies were directed to researchers, reviewing the methods used for coding tobacco-related Twitter data (91), while the others are more specific to the disease itself from the perspective of patients and health care workers. Only one review addressed the PC group which analyses the method of using the digital source to increase the efficiency of communication between the patient and the clinician (80).

**Figure 5:**
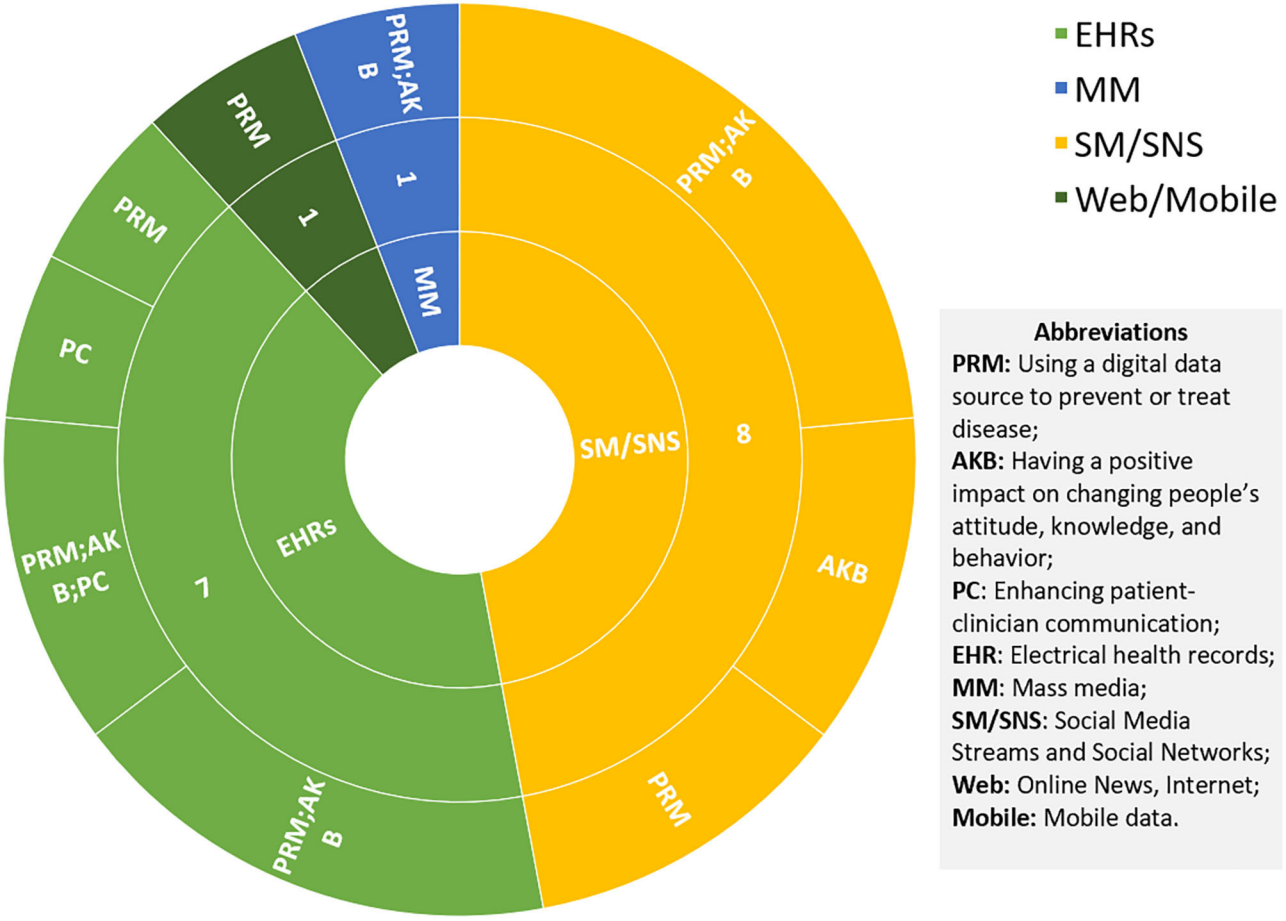
Sunburst chart of each digital source and its impact on UHC.

Social media (SM) and social networking sites (SNS) are the most popular channel for delivering interventions or knowledge to the targeted groups, as shown in Figure 5. Although they rely on interactivity, timeliness, and low cost to gain an invaluable advantage in the intervention of smoking cessation (88, 89, 91), blood glucose and pressure control (77, 79, 85, 87), and HIV antiretroviral treatment adherence (76), this also inevitably exposes their limitations in terms of both data and the platforms themselves. First, the efficacy and safety of digital interventions are unknown, not only due to the methodological limitations of randomized controlled trials such as small sample size (81) and absence of critical information (77), but also due to the nature of SM, that is, uncontrolled sharing of information leading to low data accuracy (79). Thus, proper recommendations from the clinician are necessary and crucial. Second, user engagement and retention are another major concern (88). The use of social media is entirely voluntary and most of the known data are self-reported by patients, which requires a high degree of self-awareness for people with a chronic disease such as diabetes or hypertension to intervene as designed, making the desired efficacy difficult to achieve. Therefore, many studies and clinical diagnostic follow-up reports are still needed (76, 87, 89).

The second most studied data source, EMRs, shows promising results (81) in clinical treatment of nicotine dependence, and it is also a valuable tool for integration between the patient and the health care provider (80). It could also help to improve the patient's self-management of blood and glucose control (92). However, these studies show that improvements in patients were inconsistent. Compared to the data source or digital tool, chronic disease patients benefit most from decision support tools that alert physicians about drug interactions, communication tools that keep them informed and engaged in their treatment regimens, and detailed reporting and tracking designed to inform progress (83). Therefore, the correlation between these improvements and data sources still needs to be tested by evidence.

#### Data Sources and Public Health Emergencies

**Figure 6:**
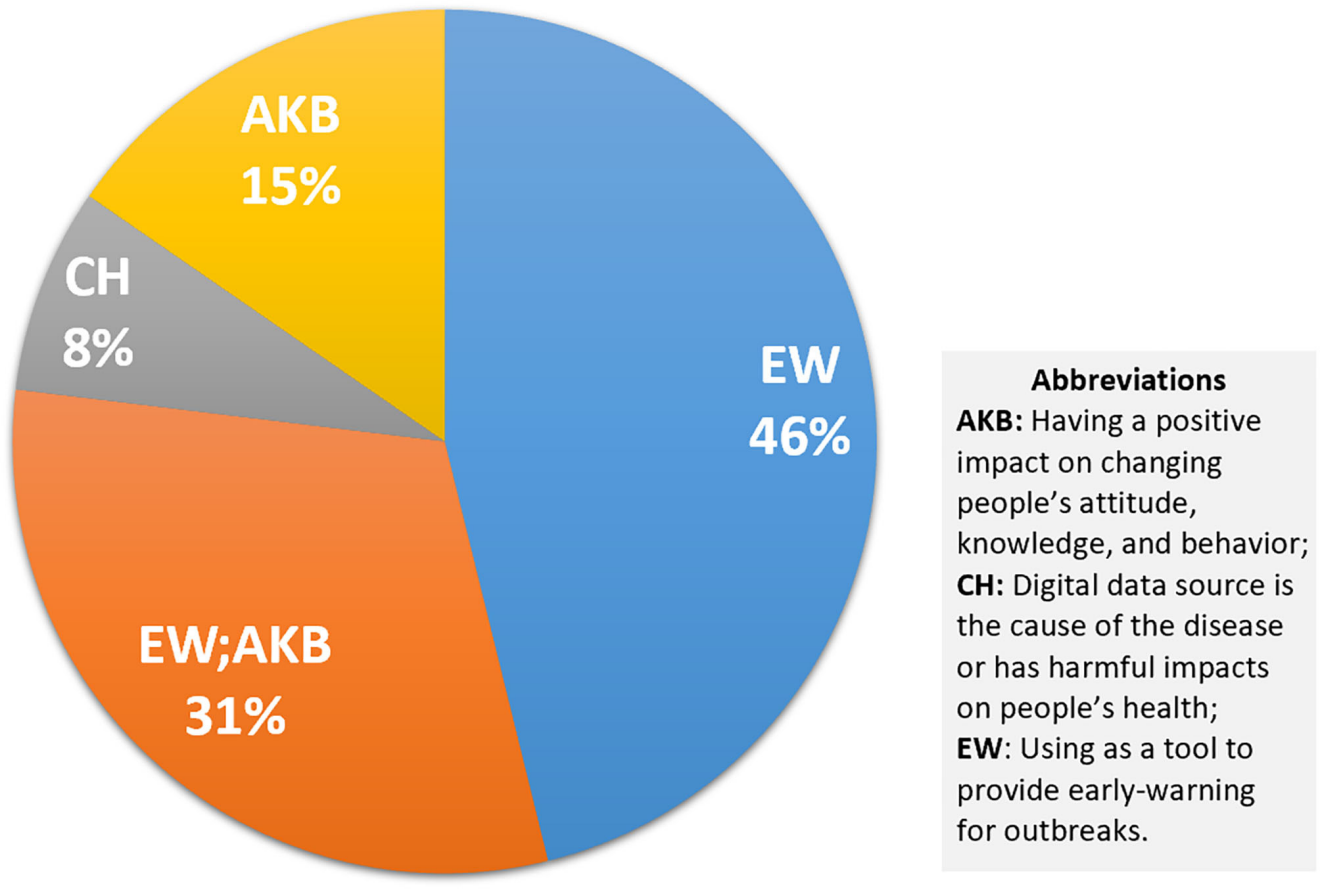
Pie chart showing the impact of digital data sources on health emergencies.

As shown in Figure 6, in the health emergency group, 77% of the 13 studies are provided for early warning, and 31% of them also look at the role of digital sources in disseminating useful information in health emergencies (93–95). Only one study is classified in the CH group, which explores the spread of health-related misinformation (96). The groups targeted by all these studies are health professionals, which includes public health experts, officials, and researchers (Supplementary Table 2).

**Figure 7:**
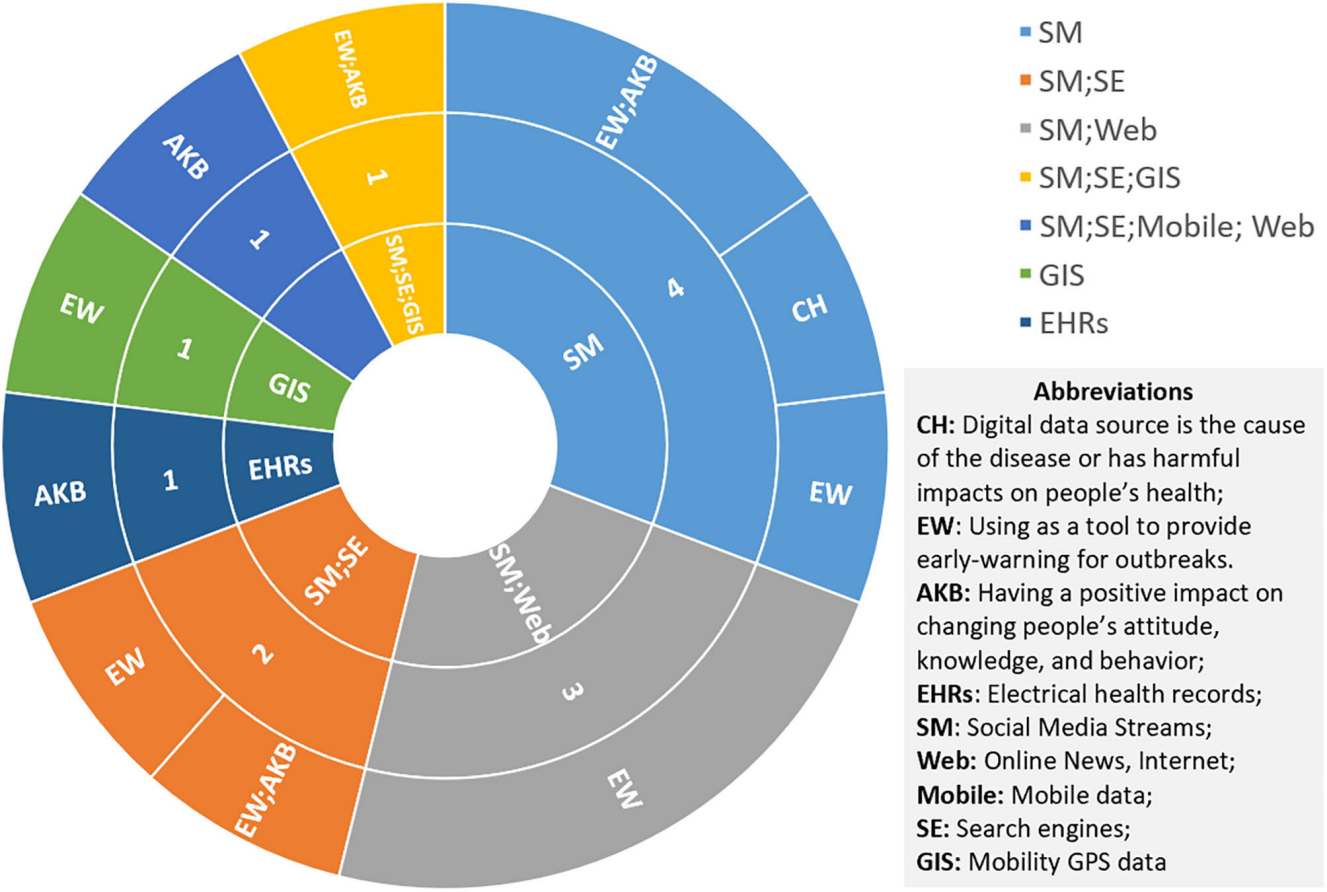
Sunburst chart displaying the impact of each digital source on health emergencies.

Social media is still a major digital data source in these reviews, which is always combined with search engines or websites (Figure 7). Most of the researchers focused on the timeliness and effectiveness of these platforms, which could provide real-time data to detect upcoming events and issue early warning to reach target audiences (54, 78). Specific data sources, including online social networks (OSNs) (24), Twitter (23), and Web GIS-based Public Health Surveillance Systems (WGPHSSs) (97), are studied. The general results reveal that adoption of these internet-based data sources facilitates monitoring of evolving epidemics (98) and obtaining epidemiological data essential for decision-making (93, 94). However, challenges such as misinformation (23, 93, 96), data reliability (94, 99), difficulty in data extraction and user privacy protection (97–99), cost of access (94, 98–100), as well as international collaboration (97) remain unsolved and require further rigorous research. It is worth mentioning that the use of EHRs to increase vaccination uptake has also been studied and may have promise, even though the secure data sharing infrastructure is the main concern raised in that paper (101).

#### Data Sources and Healthier Populations

Across the 34 articles, several general themes were the most represented in systematic reviews: mental health, nutrition, overweight, alcohol, and suicide, in descending order. Findings are summarized in Supplementary Table 3 and quantified through the tables below. Articles relevant to multiple impacts appear in multiple sections.

**Figure 8:**
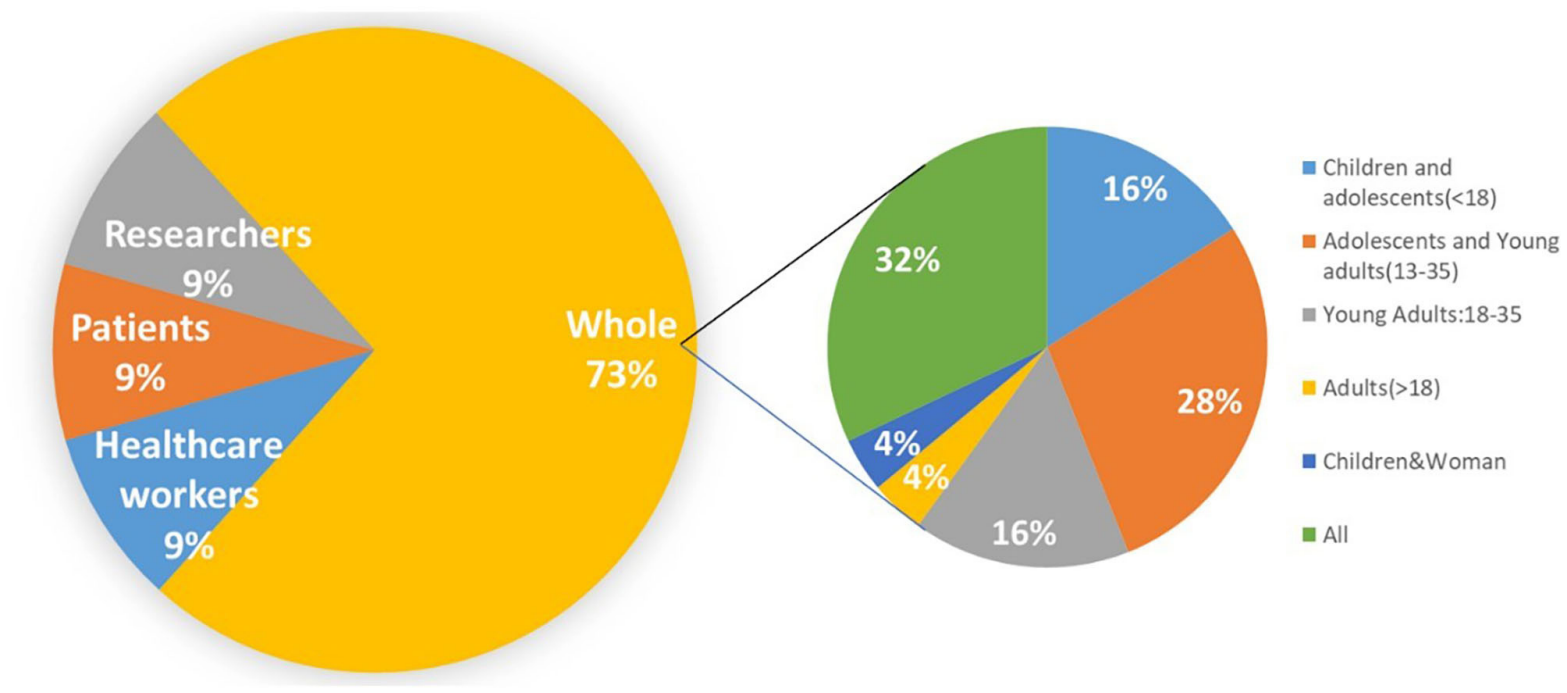
Pie chart showing targeted groups in healthier population studies.

Figure 8 shows the targeted group focused on existing studies. In addition to the disease-centered physicians, patients, and researchers, it also targets the broader human population, due to the breadth of the definition. In this population group, which accounts for 73% of the 34 studies, we break it down into age groups. Children, adolescents, and young adults appear to participate the most. This corresponds to one of the biggest limitations: younger people are the most exposed to digital data sources (102–104), which makes them privileged in benefiting from digital data, but also the most vulnerable group harmed by their shortcomings. Therefore, the results indicate that these data sources may pose a significant threat to the mental health of teenagers (104–108), potentially contributing to anxiety, depression, and even suicide (109, 110), but the strength of these associations is still uncertain and needs more controlled trials to be proven (105, 109, 111, 112).

**Figure 9:**
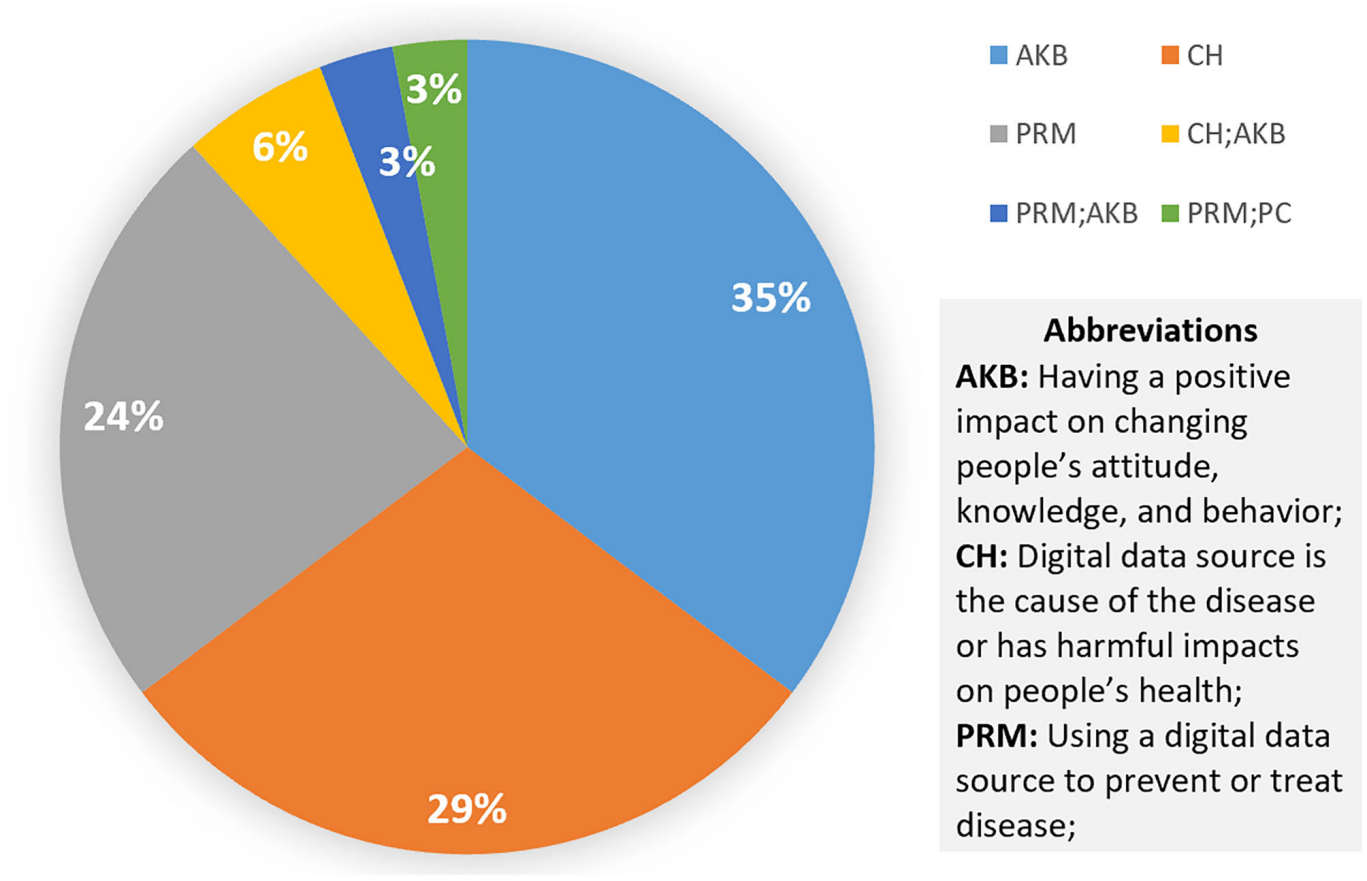
Pie chart showing the impact of digital data sources on healthier populations.

**Figure 10:**
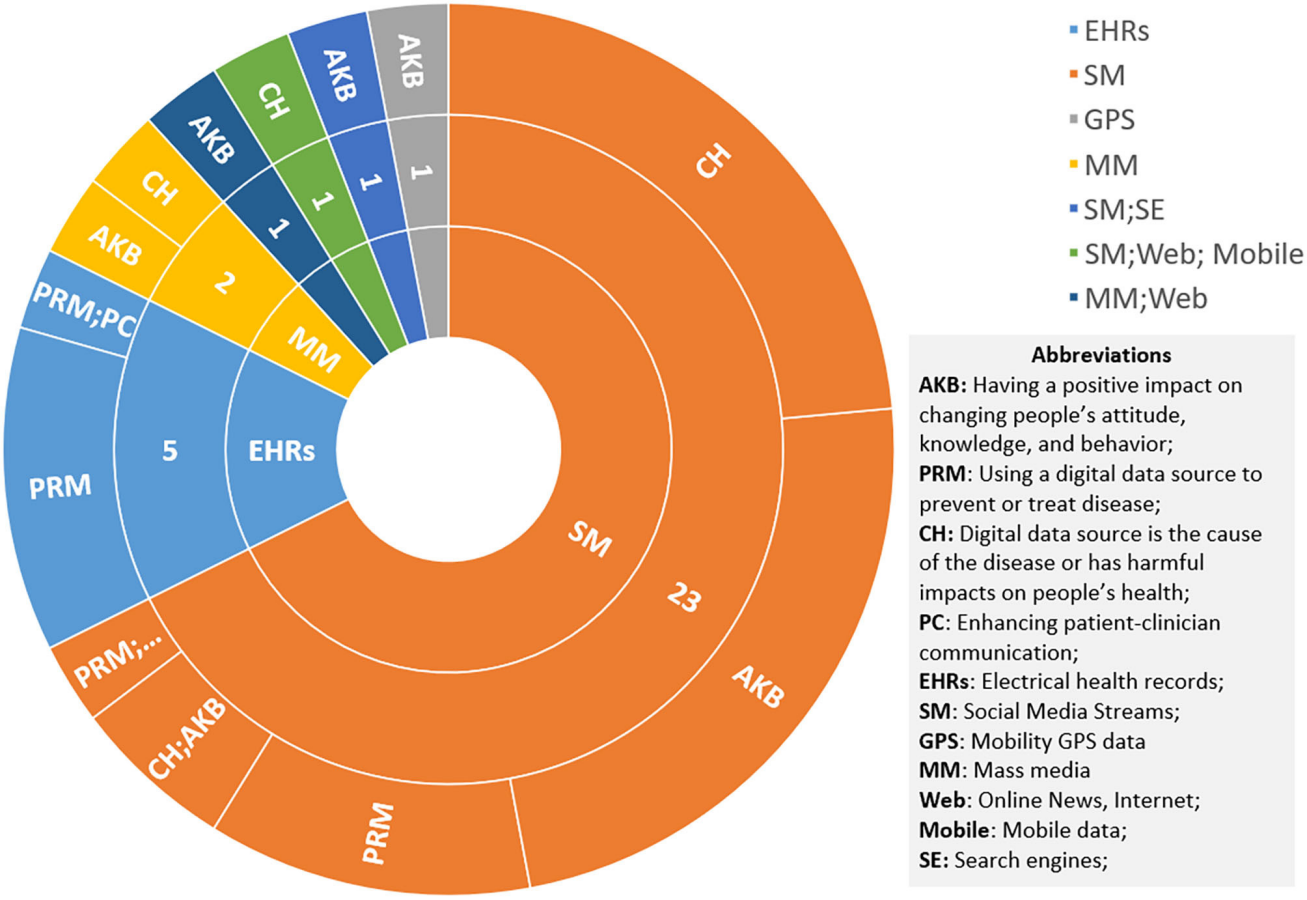
Sunburst chart displaying the impact of each digital source on healthier populations.

The most common impact areas of the digital sources from the reviews in the healthier populations' category are AKB (35%), CH (29%), and PRM (24%), as shown in Figure 9. As mentioned above, data sources can be a means of treating and intervening in chronic diseases. Here, it can also be applied to potential hazards, such as overweight (113, 114) and malnutrition (115–119). It is worth mentioning that digital sources could become a pathogenic factor. As shown by Figure 10, social media networks are the most popular source of research on the cause of disease. The most significant effect is in the area of mental health, followed by alcohol (102, 103, 120). One review argued that social media exposure contributes to young people's vulnerability to drinking by influencing their cognition or more directly affecting their drinking behavior (102). Even though social media use poses a threat in these respects, the validity of this conclusion has not been fully demonstrated (114, 121). Some studies have pointed to social media's positive intervention effect on behavior, which is more useful, engaging, and supportive than traditional measures (105, 107, 115–118).

In contrast, EHRs have a more positive role to play in this group, as shown in Figure 10. It mainly manifests in assessment, support, monitoring, and identification of high-risk people dealing with abuse (122), mental health problems (123, 124), and obesity (125). Nevertheless, since patients always underreport their experiences or symptoms and some of the high-risk groups cannot communicate (122), and technique issues result in missed diagnoses or misclassifications (122, 123), its effectiveness still needs to be enhanced by more studies (123, 126).

## Discussion and Recommendations

It is clear from the systematic review how vital the various data sources are for improving the health of populations. In this section, we will discuss the strengths and limitations of this systematic review of systematic reviews, as well as the challenges, opportunities, and recommendations for better use of data sources.

### Strengths and Limitations of the Study

The strengths of this review lie in the comprehensive and systematic approach taken, and it being the first systematic review of systematic reviews of data sources in application to the three areas highlighted in the GPW 13 and EPW along with the detailed systematic approach to review each full-text article. The evidence underpinning the data sources' impact on treatment, prevention, and control of diseases highlighted the importance of electronic health records and the increasing role of social media.

In addition to the benefits, several limitations in the review process and study design have been identified. Firstly, a significant challenge involved working to an agreed conceptualization and classification of the digital data sources. The diversification of technology has led to an increasing variability of online platforms that, while sharing some characteristics, have significant differences in design and function. Data from each platform or system might interact and play a different role for different tools, making them difficult to discuss separately (for example, GPS mobility data from GPS sensors is an input for many personal exercise apps producing GPS-tagged maps and speed/calorie output data, social media could be a data source as well as an intervention medium). These always overlap and their functionality usually includes several aspects. Thus, it is hard to produce a rigorous definition to distinguish each data source and its impact separately.

Secondly, there are a few methodological issues. This study exhibits limitations in the selecting of articles because it used only four journal databases (PubMed, IEEE Xplore, Cochrane, and Scopus), and only articles published in English, without considering gray literature. Only articles with specific data sources were included and selected, but in the keyword, the names of the popular platforms are missing, such as Twitter or Facebook, and Google search data and the health data from wearable devices (like smartwatches). Wearable devices, as a subset of IoT, have not been covered in our keywords, which might warrant a separate category in further studies. Moreover, the heterogeneity of the selected study design, methods, and outcomes may influence the results and limit the comparability of findings across studies.

Thirdly, issues were identified in the reporting of research findings. The results are difficult to quantify, but we use the number of articles as an indicator to roughly describe the amount of research in various fields. Although this is beneficial to the establishment of the overall concept, its accuracy is limited. Similarly, due to the broad scope of research, findings are potentially representative of and relevant to a wide range of populations. However, due to the cultural heterogeneity of research samples and potential issues of sampling, validity and generalizability should also be treated with caution.

Fourthly, while many mobile health (mHealth) interventions also generate data, such as mobile apps, these were excluded by our exclusion criteria to keep the systematic reviews focused and prevent the review from becoming too broad and inconclusive, and spanning the entire field of mHealth.

### Opportunities and Recommendations for Better Use of Data Sources

In this section we highlight recommendations and opportunities for further research into data sources and their impact on health outcomes.

#### Data Privacy and Transparent Data Governance and Interoperability

Digital solutions leveraging data sources require multisectoral communication, national cooperation, and collaboration between health care professionals, health service providers, and citizens to meet the essential sides of the triangle: (1) interoperability and data exchange to leverage the data sources, (2) transparent data governance, and (3) citizens and patients' understanding of the data privacy and trust in how their data are being used (62, 127).

#### Inclusivity of Data Source Design and Implementation

While systematic reviews distinguish the two groups, patients and health care professionals, digital solutions should be designed to serve all population groups and enhance the representativeness and accuracy of information. Moreover, they should grant quality monitoring by increasing their transparency and accountability and allow citizen participation and inclusion.

#### Research Into the Gap in Systematic Reviews From the Computer Science Domain

Finally, computer science research into social networks, big data streams, IoT, and sensor devices for health has been an ever-expanding discipline. However, cutting-edge computer research exploring data using artificial intelligence, machine learning, complex networks, and social computing methods is typically published at international conferences run by the Association for Computing Machinery (ACM), but there were no systematic reviews found in this database, as this form of research synthesis is not common in the field of computer science. However, conducting a systematic review of computer science-led research studies using data sources for health impact and early-warning predictive AI and social media research [for e.g., (48, 128, 129)] would be a valuable endeavor in filling this large gap.

#### Misinformation and Social Media Data Sources

The role of misinformation and its negative impact on health outcomes and mental health of citizens has been highlighted by numerous studies examining the effect of social media on body weight, nutrition, vaccination, and obesity. In particular, misinformation around vaccinations is a pressing topic (55), which is of particular importance in this time of global roll-out of COVID-19 vaccination programs (130). More research is needed to understand information spread through social media (56) in order to develop effective interventions to stop the perpetrators and break the dissemination of misinformation through closer collaboration of public health experts and social media companies.

## Conclusion

This systematic review examines the impact of digital data sources on three aspects of public health. It defines and classifies mainstream data sources and investigates studies applied to the GPW13 (2) and EPW (3) as the research framework to collect and summarize all relevant systematic reviews since 2010. The results show that social media are the most studied digital data source in defined aspects, and their primary role is in the early warning of infectious diseases and the intervention of chronic disease treatment. The second is EHRs, which have broad potential for monitoring high-risk populations and detecting potential diseases, compared to traditional paper-based data.

While the opportunities involving new data sources are growing, we also identified some limitations. Social media can be harmful to adolescent mental health and encourage alcohol and tobacco use. Furthermore, misinformation and privacy issues limit their effectiveness for early warning systems. EHRs and other data sources have been poorly researched and slow to evolve. Other data sources, such as real-time data traces, IoT streams, and mobility data, were not given enough attention due to privacy and corporate ownership challenges, and have not yet been summarized in systematic reviews due to the differences in the computer science and engineering publication models. In addition to improving data access, conducting systematic reviews of technical studies published in the ACM, IEEE, and other outlets is an area of opportunity.

To conclude, digital data sources are uniquely important for enhancing productivity, making a positive impact, and have great potential for promoting public health, but further studies are needed to enhance their strength and overcome their limitations. In particular, it is important to address trust, interoperability, and governance to leverage data sources, include computer science research in systematic studies to bridge the domain gaps, and conduct more actionable research into misinformation, in particular, on social media.

## Data Availability Statement

The original contributions presented in the study are included in the article/Supplementary Material, further inquiries can be directed to the corresponding author/s.

## Author Contributions

DN-O, NA-M, and PK: conception or design of the work and final approval of the version to be published. LL: data collection. LL and PK: data analysis, interpretation, and drafting the article. LL, PK, DN-O, and NA-M: critical revision of the article. All authors contributed to the article and approved the submitted version.

## Conflict of Interest

The authors declare that the research was conducted in the absence of any commercial or financial relationships that could be construed as a potential conflict of interest.
