# Supplementary material for: Digital Data Sources and Their Impact on People's Health: A Systematic Review of Systematic Reviews
Source: Front Public Health. 2021 May 5;9:645260. doi: 10.3389/fpubh.2021.645260 (PMC8131671; doi:10.3389/fpubh.2021.645260)
Supplement: Supplementary file 1 [file Data_Sheet_1.docx]

# Appendix 1. Keywords and Commands Table

| Keywords | |
| --- | --- |
| #1.Digital data source | ("digital epidemiology" or "digital datastream" or "digital data" or "real-time data" or "electronic medical record" or "electronic laboratory record" or "online new" or "internet media source" or "online newspaper" or "blog*" or "online media" or "online search" or "search data" or "digital trace" or "data trace" or "social media" or "social network" or "mobile data" or "gps data" or "mobility data") and ("systematic review"): ti,ab,kw |
| #2.Universal Health Coverage(UHC) | ("universal health coverage" OR UHC OR "reproductive health" OR "maternal health" OR "newborn health" OR "family planning" OR "modern contraceptive" OR "postpartum amenorrhoeic" OR "child health" OR "antenatal care" OR "pregnancy" OR "child immunization" OR "diphtheria-tetanus-pertussis" OR DTP3 OR MCV2 OR PC3V3 OR "Infectious disease" OR "tuberculosis" OR TB OR HIV OR "Human immunodeficiency virus" OR "malaria" OR WASH OR "sanitation" OR "hygiene" OR "Noncommunicable disease" OR NCD OR "cardiovascular disease" OR "blood pressure" OR "hypertension" OR "diabetes" OR "tobacco" OR "smoke" OR "cancer" OR "chronic respiratory disease" OR "chronic obstructive pulmonary disease" OR "asthma" OR "hyperglycemia" OR "hyperlipidemia" OR "risk factor" OR "service coverage" OR "essential health service" OR "hospital access" OR "hospital bed" OR "health worker" OR "health professional" OR "health security" OR "international health regulations" OR IHR): ti,ab,kw |
| #3.Health emergencies | ("emergency prepare*" OR "health emergency*" OR "emergency prevent*" OR "vaccine coverage*" OR "vaccination coverage*" OR "epidemic*" OR "pandemic*" OR "emergency vaccine*" OR "notifiable event" OR "emergency detect*" OR "emergency respond*"): ti,ab,kw |
| #4.Healthier population | ("childhood stunting" OR "childhood wasting" OR "childhood overweight" OR "suicide*" OR "alcohol*" OR "mental health*" OR "road death*" OR "road safety" OR "tobacco use" OR "partner violence" OR "gender violence*" OR "sanitation service*" OR "clean fuel*" OR "violence against children*" OR "obesity*" OR "overweight*" OR "diet*" OR "physical activity*"):ti,ab,kw |
| COMMANDS | |
| - #1 and #2 -> Results of Digital data source & UHC - #1 and #3 -> Results of Digital data source & Health emergencies - #1 and #4 -> Results of Digital data source & Healthier population | |
