# Supplementary material for: Digital Data Sources and Their Impact on People's Health: A Systematic Review of Systematic Reviews
Source: Front Public Health. 2021 May 5;9:645260. doi: 10.3389/fpubh.2021.645260 (PMC8131671; doi:10.3389/fpubh.2021.645260)
Supplement: Supplementary file 2 [file Data_Sheet_2.docx]

# Appendix 2. AMSTAR Checklist

**Table:** Assessment of Multiple Systematic Reviews (AMSTAR) Criteria:

| **Assessment of Multiple Systematic Reviews (AMSTAR) Checklist:** |
| --- |
| 1. Was an “a priori” design provided? |
| 2. Was there duplicate study selection and data extraction? |
| 3. Was a comprehensive literature search performed? |
| 4. Was the status of publication (i.e. grey literature) used as an inclusion criterion? |
| 5. Was a list of studies (included and excluded) provided? |
| 6. Were the characteristics of the included studies provided? |
| 7. Was the scientific quality of the included studies assessed and documented? |
| 8. Was the scientific quality of the included studies used appropriately in formulating conclusions? |
| 9. Were the methods used to combine the findings of studies appropriate? |
| 10. Was the likelihood of publication bias assessed? |
| 11. Was the conflict of interest stated? |
