# Supplementary material for: Digital Data Sources and Their Impact on People's Health: A Systematic Review of Systematic Reviews
Source: Front Public Health. 2021 May 5;9:645260. doi: 10.3389/fpubh.2021.645260 (PMC8131671; doi:10.3389/fpubh.2021.645260)
Supplement: Supplementary file 3 [file Table_1.docx]

| **Reference** | **AMSTAR total** | **UHC 4 categories** | **UHC 16 health services** | **Number of participants/studies** | **Study area** | **Data source** | **Use of data source** | **Impact on** | **Target group** | **Result and outcome** | **Limitations** |
| --- | --- | --- | --- | --- | --- | --- | --- | --- | --- | --- | --- |
| Carson et al., 2017 (86) | 10 | NCD | Tobacco (non-) smoking | 90 125 participants/8 studies | Norway, United States | MM | Intervention | PRM; AKB | Patient | Three out of eight studies found that mass media campaigns (television, radio, newspapers, billboards, and booklets) were effective in preventing smoking in youth. Implementation of combined school‐based components (for example, school posters) and use of repetitive media messages delivered by multiple channels (for example, newspapers, radio, television) appeared to contribute to successful campaigns. | Difficult to compare such a diverse mass media campaign over several delivery channels to draw recommendations or conclusions. |
| Alanzi et al., 2018 (87) | 5 | NCD | Prevention and treatment of raised blood glucose | 5 studies | Middle East | SM | Intervention | PRM | Patient | The reviewed studies demonstrated the potential of social networking tools being adopted in regions in the Middle East to improve the management of diabetes. | Only focusing on the Middle Eastern region by using small sample size and lack of regional diversity. |
| Frances et al., 2010 (81) | 6 | NCD | Tobacco (non-) smoking | 10 studies | United States | EHRs | Access | PRM; AKB | Health care professional | The use of EMRs to prompt or provide feedback on the clinical treatment of nicotine dependence demonstrates some promising results. | Substantial additional research is needed to understand the effects of EMRs on provider and patient behaviour. |
| Boyle et al., 2014 (82) | 8 | NCD | Tobacco (non-) smoking | 124 clinics/ 16 studies | Unknown | EHRs | Intervention | PRM | Health care professional | The review demonstrated opportunity of EHR for advice, prescribing and treatment but do not report quitting. | Link data to behaviour outcome, quitting. |
| Chan et al., 2019 (77) | 10 | RMNC | Antenatal and delivery care | 3 368 participants/16 studies | Australia, China, Indonesia, Ireland, Israel, United Kingdom, United States | SM | Intervention | AKB | Patient | The social media and mobile health (mHealth) app interventions were effective in promoting maternal physical health including weight management, control of gestational diabetes mellitus, and asthma control, with a moderate-to-large effect (d=0.72). Weight control interventions using wearable devices were more effective. | Inadequate participant information, including socioeconomic status and health status. |
| De Angelis et al., 2018 (78) | 11 | NCD | Prevention and treatment of raised blood glucose | 431 participants/7 studies | Unknown | SM | Created | PRM.AKB | Health care professional | Health professionals viewed discussion forums and collaborative projects as useful social media platforms to facilitate chronic disease self-management with patients. | The most common barrier to using social media platforms was the lack of time in health professionals’ schedules. |
| Elnaggar et al., 2020 (79) | 8 | NCD | Prevention and treatment of raised blood glucose | 2 650 participants/7 studies | Israel, Italy, Macedonia, United States | SM | Intervention | PRM; AKB | Patient | Social media help diabetes patients’ self-care based on 4 elements: (1) seeking support or encouragement from individuals with similar conditions, (2) seeking information and advice about clinical diabetes care, (3) obtaining advice about lifestyle changes, and (4) providing a sense of companionship. | Efficacy and safety are unknown in social media use among peers for diabetes self-care and other conditions. |
| Hawley et al., 2014 (80) | 9 | RMNC | Antenatal and delivery care | 43 studies | Australia, Canada, Denmark, Finland, Switzerland, United Kingdom, Zimbabwe | EHRs | Access | PC | Health care professional | In a general practice shared-care model, personal health records (PHR) and EHRs are valuable tools for integration between the patient and the health care provider. | Issues of fragmentation and continued paper use still exist. |
| Kuo et al., 2016 (92) | 7 | NCD | Prevention and treatment of raised blood glucose | 11 studies | British Columbia, China, United States | EHRs | Access | PRM; AKB | Health care professional | EHRs could help to improve patients’ hemoglobin A1c (HbA1c) using secure messaging. However, improvements in patients’ secondary outcomes, including blood pressure and cholesterol, were inconsistent. | Studies used multipronged interventions, making it difficult to establish which aspect of the intervention truly impacts patient outcomes. |
| Lessing et al., 2018 (83) | 5 | NCD | Prevention and treatment of raised blood glucose | 13 studies | United States | EHRs | Access | PRM; AKB; PC | Health care professional | Chronic disease patients benefit most by decision support tools that alert physicians of drug interactions, communication tools that keep them informed and engaged in their treatment regimens and detailed reporting and tracking designed to inform progress. | The way researchers study diseases have been influenced and medical care appears to be more integrated, the actual patient outcomes of these systems remain largely unknown. |
| Lienemann et al., 2017 (91) | 8 | NCD | Tobacco (non-) smoking | 27 studies | Unknown | SM | Mining | AKB | Researchers | Standards for data collection and coding should be developed to facilitate comparison and replication of tobacco-related Twitter results. Being relatively novel and widely used among adolescents and Black and Hispanic individuals, Twitter could provide a rich source of tobacco surveillance data among vulnerable populations. | Twitter data do not provide much information on the effects of tweets on behaviour. |
| Luo et al., 2020 (88) | 8 | NCD | Tobacco (non-) smoking | 13 studies | Canada, China, United Kingdom, United States | SM | Intervention | PRM; AKB | Patient | It highlights the effectiveness of social media–based smoking cessation interventions as social media such as Facebook, Twitter allow for increased interactions between smokers and interventionists. It also can provide tailored information and peer support. | Without biomarker verification and based only on self-reports, the cessation results generated from social media may be overestimated. |
| Naslund et al., 2017 (89) | 7 | NCD | Tobacco (non-) smoking | 9 755 participants/7 studies | Unknown | SM | Intervention | PRM | Patient | Tailored content, targeted reminders, and moderated discussions were used to promote participant engagement in smoking cessation campaigns. Posting comments or liking content may be associated with improved outcomes. | Not clear whether user engagement and retention could translate into clinically meaningful smoking cessation outcomes. |
| Osborn et al., 2010 (90) | 7 | NCD | Prevention and treatment of raised blood glucose | 2 165 participants/26 studies | Sweden, United States | EHRs | Access | PRM; PC; AKB | Patient | EPR made available over web portals enabled better communication, less distress, willingness to pay for access to care, need for patient-centric care/design and usability. | Patient privacy. |
| Riazi et al., 2015 (84) | 7 | NCD | Prevention and treatment of raised blood glucose | 51 155 participants/67 studies | Unknown | EHRs | Intervention | PRM; AKB | Health care professional | Information technology-based interventions can improve glycemic control in patients with diabetes and lead to better management of diabetes with different effect of intervention on various clinical findings. | Poor technological skills among elderly people may cause problems in using these interventions. Some patients are too busy to use them. Randomized studies are needed. |
| Toma et al., 2014 (85) | 10 | NCD | Prevention and treatment of raised blood glucose | 5 575 participants/34 studies | Canada, Finland, France, India, Iran, Israel, Italy, Korea, Taiwan, Turkey, United Kingdom, United States | SNS | Created | PRM; AKB | Health care professional | SNS offers a feasible approach to improving glycemic control, compared to standard medical management with observing significant reductions in blood pressure, triglycerides, and total cholesterol. | Unquantifiable effect on the efficacy of SNS technologies. Compliance may limit the long-term applicability of some intensive SNS interventions. |
| Wang et al., 2019 (76) | 10 | IF | HIV antiretroviral treatment (ART) | 19 studies/ 3 937 participants | Botswana, Brazil, Cameroon, China, India, Kenya, Malaysia, South Africa, Uganda, United States | Mobile; web | Intervention | PRM | Patient | eHealth interventions could be used as an effective method of increasing ART adherence of people living with HIV. | ART adherence along with long-term effectiveness of interventions, effectiveness of real-time adherence monitoring, and influences on biochemical outcomes are unknown. |
