# Supplementary material for: Digital Data Sources and Their Impact on People's Health: A Systematic Review of Systematic Reviews
Source: Front Public Health. 2021 May 5;9:645260. doi: 10.3389/fpubh.2021.645260 (PMC8131671; doi:10.3389/fpubh.2021.645260)
Supplement: Supplementary file 4 [file Table_2.docx]

| **Reference** | **AMSTER total** | **Context** | **Number of studies** | **Data source** | **Use of data source** | **Impact on** | **Target group** | **Result and outcome** | **Limitations** |
| --- | --- | --- | --- | --- | --- | --- | --- | --- | --- |
| Yan et al., 2017 (54) | 9 | General outbreaks | 84 | SM; SE | Mining; access | EW | Health professional community | Surveillance needs vary depending on the objective, and when the objective is timely epidemic detection, internet-based sources can complement and enhance traditional approaches to surveillance by using automated intelligence methods to increase efficiency. | Overlaps between systems and sources. Incomplete information provided by systems. Error and bias in text mining and natural language processing. Delay or selection bias where human moderators attended. |
| Corsi et al., 2020 (93) | 7 | COVID-19; general outbreaks | 45 | SM; SE; GIS | Mining; access | EW; AKB | Health professional community | Most addressed outbreaks: influenza and Ebola; the most recurrent types of data come from social media and search engines, being data characterized as unstructured with difficulties in treatment. | Misleading information and the result is insufficient for correct decision-making. Lack of data sharing between organizations or even countries. |
| De Araújo et al., 2020 (94) | 8 | Zika; dengue; H1N1 | 9 | SM; SE | Mining; access | EW; AKB | Health professional community | SM interactions through platforms such as Facebook, Instagram, and Twitter are tools for rapid, low-cost dissemination of information to target audiences. Facilities monitor evolving epidemics and obtain epidemiological data useful for decision-making. | Data reliability, objectivity, and relevance. Completeness and clarity of information. The dynamic aspect of networks and constant changes occur in public health settings. |
| Tang et al., 2018 (23) | 9 | H1N1; Ebola; H7N9; | 30 | SM | Mining; H7N9; | EW; AKB | Health professional community | Twitter was the most often studied social media platform, followed by YouTube, Facebook, and blogs. Three major objects: 1. assessment of the public’s interest in and responses to emerging infectious diseases; 2. examination of organizations’ use of social media in communicating emerging infectious diseases (EIDs); 3. evaluation of the accuracy of EID-related medical information. | Misinformation on social media. Diffusion of information. Practical implications from public health professionals are needed. |
| Alamoodi et al., 2020 (95) | 6 | COVID-19 | 28 | SM | Access; mining | EW; AKB | Health professional community | In the sentiment analysis and opinion mining domain in mitigating diseases, outbreaks and infectious diseases, sentiment analysis has shown its significance via four main aspects: (1) monitoring, (2) discovery, (3) news sharing and (4) policies. | Noisy nature of data from social media sites and insufficiency and data irrelevancy. SM platform reliability and authenticity. Cadence of content capability limitation. Possible exaggeration. |
| O’Shea et al., 2017 (99) | 7 | General outbreaks | 105 | SM; Web | Access; mining | EW | Health professional community | There is an increase in internet data source diversity in newly identified systems. Event-based internet biosurveillance may supplement traditional systems for a more comprehensive estimate of disease burden, | Data reliability. Difficulty differentiating signal from noise. Lack of specificity and results from these systems differ from official sources. Individuals’ privacy. Cost of access. |
| Al-garadi et al., 2016 (24) | 10 | General outbreaks | 20 | SM | Access; mining | EW | Health professional community | Online social networks offer complementary data that work best when integrated with traditional data. Collecting representative data with a sufficient population coverage, which leads to the lack of well-defined study population, remains a challenge. | User selection bias and keyword selection bias. Not interpreted by specialists for relevance. Data posted in social network websites are difficult to verify. The collection of representative data of sufficient population coverage and in the lack of a well-defined study population. |
| Wang et al., 2019 (96) | 6 | Vaccine; Ebola; Zika | 57 | SM | Access; mining; created | CH; PRM | Health professional community | Misinformation is highly prevalent on social media and tends to be more popular than accurate information, while its narrative often induces fear, anxiety, and mistrust of institutions. | Meanings of misinformation and “fake news” by authors can vary. It is very difficult to ascertain the motives of those spreading particular rumours and myths and to distinguish hard or bad messages. Misinformation about other issues can have health consequences, which is not included in this study. |
| Velasco et al., 2014 (98) | 6 | General outbreaks | 13 | SM; Web | Access; mining | EW | Health professional community | The dissemination to health authorities of new information about health events is not efficient and could be improved. No comprehensive evaluations show whether event‐based surveillance systems have been integrated into actual epidemiological work during real‐time health events. | Data protection and privacy. Strategy to compare and cross‐verify indicator‐based and event‐based data. Time‐consuming and costly collaboration between professionals. |
| Barros et al., 2020 (136) | 10 | General outbreaks | 162 | SM; Web | Access; mining | EW | Health professional community | The knowledge gathered from this review suggests that search queries and social media provide useful data for monitoring infectious diseases. In terms of studying chronic illnesses, discussion forums and social media are preferred. | Research gaps: new terminologies crucial for the detection of previously targeted diseases will be missed. Strong susceptibility to media events and the absence of approaches dealing. Absence of consistent training and test periods. |
| Luan et al., 2014 (97) | 8 | General outbreaks | 39 | GIS | Access | EW | Health professional community | Use of news and social media and web user search records as data sources, participatory public health surveillance, collaborations among health sectors at different spatial levels and among various disciplines, adaption or reuse of existing Web GIS-Based Public Health Surveillance Systems, and adoption of geohash and open-source development models were identified as the directions for advancing WGPHSSs. | Integrating health-related data from multiple sources and enhancing the data analysis functions in WGPHSSs to improve their performance. International collaboration. Data privacy. Regional inequality. |
| Odone et al., 2015 (100) | 8 | Vaccine | 19 | Mobile; SM; Web; SE | Intervention | AKB, PRM | Health professional community | Although there is great potential for improving vaccine uptake and vaccine coverage by implementing programmes and interventions that apply new media, scant data are available and further rigorous research is needed. | The effectiveness and cost-effectiveness of interventions applying new media. Need to know how to successfully market constructive public health messages. |
| Balzarini et al., 2020 (101) | 9 | Vaccine | 8 | EHRs | Intervention | AKB | Health professional community | Evidence suggests a moderate positive impact of personal electronic health records (PEHRs) access in increasing vaccine uptake, with data available for influenza and pneumococcal vaccines, diabetic patients and childhood immunization. Pooled data report the addition of digital communication features might increase vaccine uptake, compared to PEHR access alone. | None of PEHRs platforms described in this review included or focused on mobile technology extensions. Need to ensure adequate digital literacy in different settings. Secure data sharing infrastructure is required. |
