# Supplementary material for: Digital Data Sources and Their Impact on People's Health: A Systematic Review of Systematic Reviews
Source: Front Public Health. 2021 May 5;9:645260. doi: 10.3389/fpubh.2021.645260 (PMC8131671; doi:10.3389/fpubh.2021.645260)
Supplement: Supplementary file 5 [file Table_3.docx]

| **Reference** | **AMSTER total** | **Context** | **Number of participants/studies** | **Study area** | **Data source** | **Use of data source** | **Impact on** | **Target group** | **Result and outcome** | **Limitations/Unknown** |
| --- | --- | --- | --- | --- | --- | --- | --- | --- | --- | --- |
| Curtis et al., 2018 (102) | 8 | Alcohol | 10 295 participants /19 studies | Australia, Belgium, Netherlands, United States | SM | Intervention | CH | Adolescents and young adults | Greater alcohol‐related SM engagement correlated with both greater self‐reported drinking and alcohol‐related problems. | Whether SM exposure contributes to young people’s vulnerability to drinking by influencing their cognition or more directly affecting their drinking behaviour. How to use SM‐based interventions aimed at reducing heavy drinking and alcohol‐related problems. |
| Syed et al., 2020 (122) | 10 | Violence | 3 875 183 participants/88 studies | United States 81%, Australia 9% Europe 4% | EHRs | Access | PRM; PC | Children; women | Coded indicators in EHRs have a high likelihood of correctly classifying types of child maltreatment (CM) and intimate partner violence (IPV) across the life course, providing a useful tool for assessment, support, and monitoring of high-risk groups in health services and research. | Missed diagnoses or misclassifications: patients often underreport their experiences or symptoms, and high-risk groups cannot communicate. Some symptoms addressed by the reference standard may not have been conveyed to the clinician. |
| Noel et al., 2020 (103) | 9 | Alcohol | 25 studies | Australia, Brazil, China, New Zealand, Thailand, United Kingdom, United States | SM | Intervention | CH | Adolescents and young adults | Engagement with digital alcohol marketing (for example, clicking on an alcohol ad, visiting an alcohol-branded website, liking or sharing an ad on SM, or downloading alcohol-branded content) is positively associated with increased alcohol consumption and increased binge or hazardous drinking behaviour. | Multiwave prospective cohort studies should allow for the construction of path models that can test a marketing receptivity to attitudes to behaviour onset causal chain and test for any reciprocal effects between digital alcohol marketing practices, attitudes, and behaviours. |
| Jamnadass 2018 (133) | 6 | Kidney stone disease (KSD) | 10 studies | European countries | SM; SE | Mining; access | AKB | Patients | SM and search engines provide valuable information to patients with KSD. | Not comprehensive enough to include advice on other aspects of KSD prevention. |
| Larvin et al., 2019 (123) | 11 | CMD | 22 107 participants /14 studies | Canada, Netherlands, Spain, Sweden, United Kingdom, United States | EHRs | Access | PRM | Health care workers | Case definitions effectively identify cases in a population with good accuracy and few false positives. Sensitivity is more variable and specificity is higher in depressive cases. Adding context to case definitions may improve overall case-finding accuracy. | Routine primary care databases used in the present review may have variable accuracy. Developments need to improve concordance of EHR coding. |
| Piteo et al., 2020 (105) | 11 | Mental health | 34 794 participants /19 studies | Australia, Belgium, Hungary, Thailand, United States | SNS | Intervention | CH | Children and adolescents: aged 5 to 18 | There are positive associations between Social Network (SNS) use, including the amount of time spent on SNS, passive use of SNS, addictive and problematic SNS behaviours and depressive symptoms. There are also positive associations between time spent on SNS and investment in SNS use and anxiety symptoms. | While some of these studies have begun to examine the mediating factors that may contribute to or exacerbate the proposed relationship, there are several underexplored factors that may mediate this relationship. |
| Carreira et al., 2019 (124) | 8 | Mental health | 120 studies | United Kingdom | EHRs | Access | PRM | Patients | There was substantial variability in the code lists of mental health. There is a need for standardized definitions and validated list of codes to assess mental health and quality-of-life outcomes in primary care databases in the United Kingdom. | Transparency in the list of codes. Validation of the outcomes. |
| Koleck et al., 2019 (126) | 8 | General health | 27 studies | Unreported | EHRs | Access | PRM | Health care workers | Natural language processing is used to extract information from EHR free-text narratives written by various health care providers on a wide range of symptoms across diverse clinical specialties. The current focus of this field is on the development of methods to extract symptom information and the use of symptom information for disease classification tasks rather than the examination of symptoms themselves. | Need accurate extraction of severity and other contextual factors. Need more investigations focused on symptoms and symptom documentation as well as symptom management as primary outcomes of interest from the free-text narratives of EHRs in addition to studies on the use of symptom information to characterize disease or predict response to treatment. |
| Karim 2020 (121) | 5 | Mental health | 16 studies | Unreported | SM | Intervention | CH | All | SM affect the level of anxiety and depression in individuals. Other factors such as interpersonal trust and family functioning may have a greater influence on the symptoms of depression than the frequency of SM use. | Further investigations are required to clarify the underlying factors that help examine why SM have a negative impact on some peoples’ mental health, and no effect or a positive effect on the mental health of others. |
| Sharma et al., 2020 (106) | 11 | Mental health | 12 989 participants/16 studies | Unreported | SM | Intervention | CH | Young people | Users being more influenced by the feedback they received and would rather stay online and socialize than partake in offline activities. The detrimental effects observed in a user’s sense of well-being are feelings of meaninglessness and a lower level of state and trait self-esteem and self-evaluation. | Still need a greater number of randomized controlled trials (RCTs) conducted to measure various variables amongst different age groups. Longitudinal research was conducted. |
| Karmegam et al., 2019 (134) | 10 | Mental health | 18 studies | Unreported | SM | Mining; access | PRM | Patients | Information extracted from SM data provides valuable information about the emotions of the population during and after disasters and augments the traditional methods of information gathering at the time of a disaster. The data collected also aid the public health professionals on the response team in decision-making. | Only younger adults and the socioeconomically privileged. Data are not representative of all Twitter activity because of Twitter Application Programming Interface (API) limitations. Possibility of no connection between the disaster location and the person who tweets. Incomplete due to language and format limitations (links were eliminated). |
| Ridout et al., 2018 (107) | 8 | Mental health | 9 studies | Australia, China, United States | SNS | Intervention | AKB | Young people | Young people find SNS–based interventions highly usable, engaging, and supportive. | Moderation by clinical experts was identified as a key component of the more successful interventions. |
| Wongkoblap et al., 2017 (139) | 8 | Mental health | 48 studies | Unreported | SM; SNS | Mining | PRM; AKB | Researchers | Assembling large, high-quality datasets of SM users with mental disorders is problematic, not only due to biases associated with the collection methods, but also in terms of managing consent and selecting appropriate analytics techniques. | Reliability of the social network data provided; the general desirability of such interventions; the ethical issues. |
| Seabrook et al., 2016 (108) | 5 | Mental health | 34 512 participants /70 studies | Unreported | SNS | Intervention | CH | Young people | SNS use related to less loneliness and greater self-esteem and life satisfaction. Different patterns in the way individuals with depression and individuals with social anxiety engage with SNSs are beginning to emerge. | Need leveraging real-time SNS data over time, not predominantly focused on cross-sectional self-report approaches. |
| Baker et al., 2016 (111) | 8 | Mental health | 35 044 participants /30 studies | Australia, Greece, Korea, Philippines, Turkey, Serbia | SM; SNS | Intervention | CH; AKB | Young people | The relationship between online social networking and symptoms of depression may be complex and associated with multiple psychological, social, behavioural, and individual factors. | Hard to agree the conceptualization of online social networking due to its diversity. Cultural and geographical variation. |
| Rahman et al., 2020 (137) | 9 | Mental health | 22 studies | China, Greece, Japan, United States and others | SM/SNS | Mining | PRM: disease detection | Researchers | Big data in online social networks (OSNs) contribute to mental health problem detection. Mental health problem detection through OSNs necessitates comprehensive adoption, innovative algorithms, and computational linguistics to describe its limitations and challenges. | Need referrals from mental health specialists as subject matter experts are also required to help obtain accurate and effective information. |
| Keles et al., 2019 (104) | 9 | Mental health | 21 231 participants /13 studies | Unreported | SM | Intervention | CH | Adolescents and young adults | Key findings of the studies included were divided into four categories of exposure to SM: time spent; activity; investment; and addiction. All these categories were found to correlate with depression, anxiety, and psychological distress, with an acknowledgement of the complexity of these relationships. | Underexplored mediators and moderators. Unclear causality was due to the cross-sectional study design used in almost all studies and the lack of comparison group in the cohort study. |
| Yadav et al., 2015 (120) | 10 | Alcohol | 19 studies | Australia, New Zealand, United States | MM | Intervention | CH | All | No evidence of media campaigns reducing the risk of alcohol-related injuries or fatalities. | More studies are needed, including studies evaluating newly emerging media and cost-effectiveness of media campaigns. |
| McCrae et al., 2017 (132) | 6 | Mental health | 13 532 participants /11 studies | Australia, Belgium, Canada, China, Netherlands, Romania, Spain, United States | SM | Intervention | CH | Children and adolescents: aged 5 to 18 | Analysis revealed a small but statistically significant correlation between SM use and depressive symptoms in young people. However, studies varied widely in methods, sample size and results, making the clinical significance of these findings nuanced. | Researchers have lacked consensus on the phenomena for investigation, resulting in limited replication. |
| Frost et al., 2017 (112) | 9 | Mental health | 65 studies | Australia, India, Turkey, United States and others | SM | Intervention | CH | Adolescents and young adults | Facebook use was associated with six outcomes: addiction, anxiety, depression, body image, alcohol use, and other problems. The strength and validity of these relationships varied. Facebook use is a multidimensional construct. Important to assess specific SNS platforms or similar functions over platforms. | Relationships needs to be quantified and the precipitating and/or maintaining effects should be clarified, particularly in conjunction with pertinent moderators and individual difference factors. |
| Twomey et al., 2017 (138) | 9 | Mental health | 21 studies | Unreported | SM | Access | PRM: disease detection | All | Significant self-presentation associations were yielded for self-esteem, perceived social support, social anxiety, well-being, depression, bipolar/mania, stress, self-consciousness, and insecure attachment. Significant associations were also yielded for all the big five personality variables and narcissism. | None. |
| Baer et al., 2013 (125) | 9 | Overweight | 11 studies | Unreported | EHRs | Access | PRM | Health care workers | EHR interventions can increase diagnosis and treatment of overweight and obesity, but it is unclear whether this will lead to better patient outcomes. Further studies are needed to evaluate the effects of more sophisticated EHR features that assist clinicians with management, rather than merely assessment, of overweight and obesity. | First, most EHR interventions have focused on increasing identification of overweight and obesity, and few to date have included features to assist providers with management of overweight and obesity. Focus was only on clinician performance outcomes and documentation of counseling or treatment, and did not examine any patient outcomes. |
| Sisask et al., 2012 (109) | 7 | Suicide | 56 studies | Australia, China, Japan, United Kingdom, United States | MM; web | Intervention | AKB | All | Most of the studies support the idea that media reporting and suicidality are associated. However, there is a risk of reporting bias. More research is available about how irresponsible media reports can provoke suicidal behaviours (the Werther effect) and less about the protective effect the media can have (the Papageno effect). A strong modelling effect of media coverage on suicide is based on age and gender. Media reports are not representative of official suicide data and tend to exaggerate sensational suicides. | The internet is a global medium, which makes it difficult to determine the specific area and exact dose of media reporting. |
| Krenn et al., 2011 (140) | 8 | Physical activity | 24 studies | Unreported | GPS | Access | AKB | Researchers | GPS is a promising tool for improving understanding of the spatial context of physical activity. The current findings suggest that choosing the right device and efforts to maximize participant adherence are key to improving data quality, especially over longer study periods. | Data loss caused by signal dropouts, loss of device battery power, and poor adherence of participants to measurement protocols. |
| Chau et al., 2018 (115) | 9 | Nutrition | 21 977 studies /16 studies | Australia, Brazil, Malaysia Mexico, Spain, United States | SM | Intervention | AKB | Adolescents and young adults | Most of the studies identified had positive outcomes. We found that most studies used only basic SM features, did not evaluate the efficacy of SM components, and did not differentiate between the efficacy of SM and other delivery mechanisms. | Unable to separate the impact of SM from the impact of other features; SM use in the included studies was conservative and engagement with SM was limited due to the behavioural challenges and reminders. |
| Hsu et al., 2018 (116) | 10 | Nutrition | 3 554 participants /7 studies | Mexico, Portugal, Spain, United States | SM | Intervention | AKB | Adolescents | The most common improvement concerned fruit or vegetable intake, and two of four studies showed improvement in sugar-sweetened beverage consumption. The most common behaviour change techniques (BCT) used was social support, followed by demonstration of behaviour, self-monitoring, goal setting, and feedback. | Better-quality interventions, full description of the BCTs, long-term follow-up, and popular contemporary SM platforms to build the evidence base are required. |
| Nour et al., 2017 (117) | 11 | Nutrition | 3 732 participants /17 studies | Australia, United States | SM | Intervention | AKB | Young adults: aged 18–35 | SM and gaming offer a new dimension for nutrition interventions, with the current body of evidence indicating potential positive impacts on improving knowledge and attitudes. However, the implications of SM and gaming strategies in the longer-term and for influencing behaviour and health outcomes could not be determined. | The complex nature of measuring engagement with SM content is acknowledged in the literature, especially those participants who view shared content without actively engaging with it. |
| Klassen et al., 2018 (118) | 8 | Nutrition | 3 579 participants /21 studies | Australia, Brazil, United Kingdom, United States | SM | Intervention | AKB | Young adults: aged 18–36 | Interventions had a positive statistically significant impact on nutritional outcomes in 1 in 9 trials. Engagement with the SM component of interventions varied, from 3% to 69%. Young adults appear to be open to receiving healthy eating and recipe tips through SM, but they are reluctant to share personal weight-related information on their online social networks. | Concerns about public SM use is a contributing factor to poor engagement; Young people are reluctant to share personal weight-related information with their online social networks. |
| Graziose et al., 2018 (119) | 9 | Nutrition | 18 studies | Low- and middle-income countries (LMIC) | MM | Intervention | AKB | Parents | Fifteen studies reported improvements in breastfeeding and/or complementary feeding practices, using indicators recommended by the WHO, and six studies reported improvements in related psychosocial factors. However, little detail was provided on the use of formative research, a formal behaviour change theory and behaviour change techniques. Few studies reported both dose delivered and participants’ exposure to the intervention. | Concerns of review’s timeliness. The lack of published information describing intervention components. Need high consistency and replicability of Impact of Infant and Young Child Feeding (IYCF) intervention studies. |
| Chang et al., 2013 (113) | 10 | Overweight | 10 205 participants/20 studies | Unreported | SM | Intervention | AKB | Adult | Despite the widespread use of SM, few studies have quantified the effect of SM in online weight management interventions, so their impact is still unknown. Although SM may play a role in retaining and engaging participants, studies designed to measure their effect are needed to understand whether and how SM may meaningfully improve weight management. | Stranger phenomenon (SM are primarily used for the conversion and maintenance of existing relationships but not for making new acquaintances); the components designed for weight management studies may not have the same usability, access, or appeal as normal SM. |
| Holland et al., 2016 (114) | 5 | Overweight | 7 635 participants /20 studies | Australia, Germany, Netherlands, United States | SNS | Intervention | CH; AKB | Adolescents and young adults | The use of SNSs is associated with body image and disordered eating. Specific SNS activities, such as viewing and uploading photos and seeking negative feedback via status updates, were identified as particularly problematic. A small number of studies also addressed underlying processes and found that appearance-based social comparison mediated the relationship between SNS use and body image and eating concerns. Gender was not found to be a moderating factor. | Need to clarify the role played by SNS use in body image and disordered eating. |
| Williams et al., 2014 (135) | 9 | Overweight; physical activity | 22 studies | United States 68%, Australia 18% | SM | Intervention | AKB | All | Most of the current research on social media as an intervention to affect healthy diet and exercise has been conducted in the United States with overweight or obese adult populations. It is most often used as a part of complex interventions that employ other techniques such as learning modules, online self-report journals and even in-person support. | Low levels of participation and no significant differences between groups in key outcomes. |
| Mita et al., 2016 (131) | 8 | Overweight; physical activity | 10 711 participants /16 studies | Australia, Belgium, Canada, United States | SM | Intervention | AKB | All | Trials assessing SM interventions aimed at modifying risk factors (physical activity, body weight, and fruit and vegetable intake) for noncommunicable diseases showed that SM use improved primary outcomes, but the overall quality of the studies limits the generalizability of these findings. | Information accuracy, loss of reputation of health care providers, and privacy breaches. Further trials are warranted, especially to isolate the effect of SM use and to fully evaluate the effect of the social presence and media richness of SM platforms. |
| Robinson et al., 2016 (110) | 7 | Suicide | 30 studies | Unreported | SM; Web | Access | PRM; AKB | All | Suicide-related SM research describes the development of SM sites designed for suicide prevention, examining the potential of their ability to reach or identify people at risk of suicide, the ways in which people used it for suicide prevention purposes and the experiences of people who had used SM sites for suicide prevention purposes. No intervention studies were identified. | Difficulties controlling user behaviour and accurately assessing risk, issues relating to privacy and confidentiality and the possibility of contagion. |
